# Insights gained from single-cell analysis of immune cells in tofacitinib treatment of Vogt-Koyanagi-Harada disease

**DOI:** 10.1172/jci.insight.162335

**Published:** 2022-12-08

**Authors:** Xiuxing Liu, Qi Jiang, Jianjie Lv, Shizhao Yang, Zhaohao Huang, Runping Duan, Tianyu Tao, Zhaohuai Li, Rong Ju, Yingfeng Zheng, Wenru Su

**Affiliations:** State Key Laboratory of Ophthalmology, Zhongshan Ophthalmic Center, Sun Yat-sen University, Guangdong Provincial Key Laboratory of Ophthalmology and Visual Science, Guangzhou, China.

**Keywords:** Autoimmunity, Therapeutics, Cellular immune response

## Abstract

Vogt-Koyanagi-Harada disease (VKH) is an important refractory uveitis mediated by pathological T cells (TCs). Tofacitinib (TOFA) is a JAK- targeted therapy for several autoimmune diseases. However, the specific pathogenesis and targeted therapeutics for VKH remain largely unknown. Based on single-cell RNA sequencing and mass cytometry, we present what we believe is the first multimodal, high-dimensional analysis to generate a comprehensive human immune atlas regarding subset composition, gene signatures, enriched pathways, and intercellular interactions of VKH patients undergoing TOFA therapy. Patients with VKH are characterized by TCs’ polarization from naive to effector and memory subsets, together with accrued monocytes and upregulated cytokines and JAK/STAT signaling pathways. In vitro, TOFA reversed Th17/Treg imbalance and inhibited IL-2–induced STAT1/3 phosphorylation. TOFA alleviated VKH symptoms by restoring pathological TCs’ polarization and functional marker expression and downregulating cytokine signaling and lymphocyte function. Remarkably, inflammation-related responses and intercellular interactions decreased after TOFA treatment, particularly in monocytes. Notably, we identified 2 inflammation- and JAK-associated monocyte subpopulations that were strongly implicated in VKH pathogenesis and mechanisms involved in TOFA treatment. Here, we provide a potentially novel JAK-targeted therapy for VKH and elaborate on the possible therapeutic mechanisms of TOFA, expanding our knowledge of VKH pathological patterns.

## Introduction

Uveitis is the leading cause of vision loss, accounting for an estimated 25% of all blindness cases in the Western and the developing world (1, 2). Vogt-Koyanagi-Harada disease (VKH) is among the most important types of uveitis, characterized by rapid onset, recurrent inflammation, and multiple systems’ involvement (3). Besides binocular involvement and hearing loss, VKH affects the brain and spinal cord, is involved in alopecia, and causes headache (4–6). Currently, patients with VKH are primarily treated with systemic and topical glucocorticoids with or without immunosuppressive agents. Although high-dose systemic corticosteroids remain the gold standard therapy, refractory cases may require alternative agents (7). Furthermore, conventional treatment with early, high-dose, systemic corticosteroids is insufficient to prevent chronicity and vision-threatening complications, including cataracts, glaucoma, and even blindness, which have been reported in the chronic recurrent phase (8). In addition, VKH patients with osteoporosis, severe diabetes, hypertension, and glucocorticoid intolerance have limited access to effective therapy. Therefore, exploring effective and safe medications for patients with VKH is necessary.

Although previous studies have shown that immune cells participate in the autoimmune destruction of melanocyte-enriched organs in VKH, the major immune cells and inflammatory pathways involved in VKH remain poorly understood. During immune dysfunction, antigens in eye tissues can be identified, resulting in immunoreaction induced by pathological TCs, such as Th17 and Th1, and related cytokines (9). For example, IFN-γ–secreting Th1 and IL-17–secreting Th17 cells accumulate in the blood, aqueous fluid, and other tissues of patients with VKH (4, 10). Additionally, lymphocytic infiltration, B cell (BC) chemoattractant, and autoantibodies have been detected in aqueous humor (11, 12). In addition, inflammatory cytokines, such as IL-2, IL-6, IL-23, and IFN-γ, can induce T cell (TC) proliferation and the differentiation of BCs and TCs into effector and pathogenic types. These results indicate a key role of inflammatory cytokines and lymphocyte activation in VKH development. Among the cytokine-induced downstream pathways, JAKs are widely involved because they are shared by members of the receptors. Upon binding to cytokine receptors, JAKs are activated and transduce signals through phosphorylation of downstream molecules, such as STAT and other transcription factors, to trigger immune responses. IL-2 is vital in maintaining and activating lymphocytes. Genetic evidence has connected the association of JAK1/3 and IL-2 with lymphocyte proliferation and immune homeostasis (13). A knockin mouse model study indicated that the Tyk2 P (a JAK family member) allele could effectively shield animals from the disease by altering Th1 and Th17 signaling (14). STAT3 is vital for Th17 TCs’ polarization (15), and the deletion of STAT3-related genes reduces pathogenic Th17 TCs’ proliferation and cytokine production (16). However, the role of JAK/STAT signaling in VKH pathogenesis is still poorly understood, indicating that the global and explicit demonstration of immune interactions in VKH remains unfulfilled.

As a JAK-targeting inhibitor, TOFA (tofacitinib, Pfizer, Inc.) is a US FDA-approved oral medication for rheumatoid and psoriatic arthritis and ulcerative colitis (17, 18). Evidence indicates that anti-JAK targeting can downregulate the JAK/STAT signaling pathway in TCs and BCs and reduce the inflammatory response (19). TOFA has been implicated in inhibiting TCs’ activation by downregulating cytokines produced by Th17 TCs during chronic intestinal inflammation (20). Another study further supported the role of TOFA in suppressing culprit-induced TCs’ proliferation in vitro and the JAK/STAT pathway in mediating adverse drug reactions (21). TOFA suppresses pathogenic immune responses in vessel vasculitis and minimizes CD4^+^CD103^+^ tissue-resident memory TCs with minimal production of IFN-γ, IL-17, and IL-21 (22). However, the therapeutic effects of TOFA on VKH and the molecular and cellular mechanisms of JAK blockade in the human immune system remain unclear. Previous approaches are largely restricted to well-established antibody panels or sorted cell samples based on prior knowledge and, therefore, preclude the comprehensive characterization of TOFA-induced immune modulation in inflammatory diseases. Unbiased high-throughput single-cell RNA sequencing (scRNA-Seq) provides unique opportunities to gain insights into molecular mechanisms, allowing researchers to explore the overall remodeling effects of targeted therapy in drug-induced hypersensitivity syndrome (21). Thus, it was desirable to establish a comprehensive atlas of immune modulation to facilitate our understanding of the therapeutic mechanism of TOFA treatment and provide an alternative approach to personalized medicine.

Therefore, we connected mass cytometry with time of flight (CyTOF) and scRNA-Seq to generate a proteomic and transcriptomic landscape of peripheral blood mononuclear cells (PBMCs) from VKH patients. Furthermore, we compared blood immune cell properties before and after TOFA treatment to explore the therapeutic mechanisms. Our study is the first to our knowledge to treat VKH patients with TOFA to provide a comprehensive profile of its effects on the immune system.

## Results

### TOFA treatment effect on VKH symptoms and pathological TC subset ratio.

**Figure 1:**
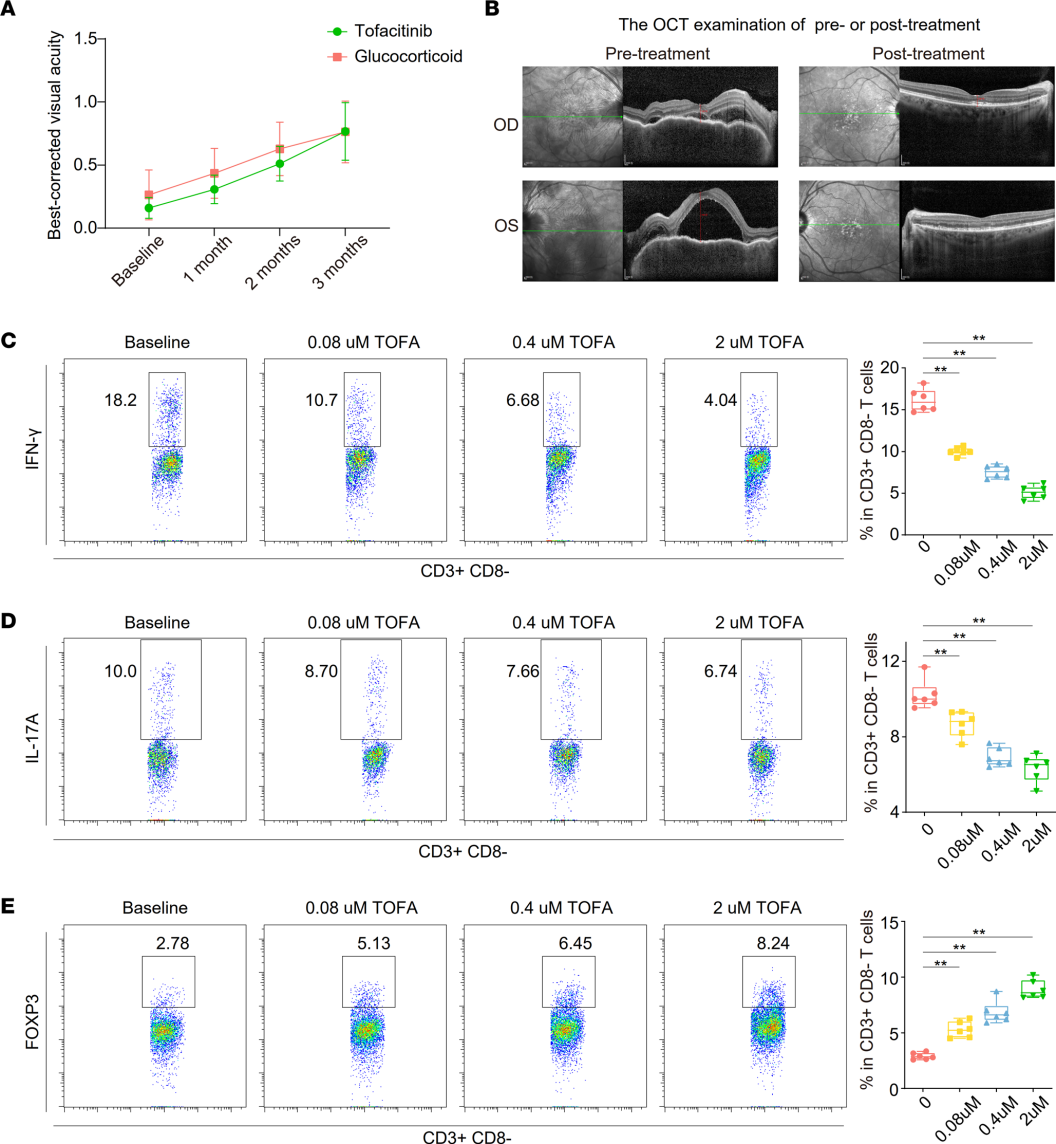
The therapeutic effect of TOFA treatment for VKH and the inhibitory effects of TOFA on PBMCs in vitro. (**A**) The BCVA from baseline to 1, 2, and 3 months after using systemic TOFA (10 individuals, 20 eyes) and glucocorticoid (18 individuals, 36 eyes). (**B**) The representative OCT examination of pretreatment or 3 months posttreatment. The flow cytometry histograms (left) and box plots (right) showing the percentage of IFN-γ (**C**), IL-17A (**D**), and FOXP3 (**E**) in CD3^+^CD8^–^ TCs (*n* = 6/group). Box plots show the interquartile range (box), median (line), and minimum and maximum (whiskers). Significance in **C**–**E** was calculated using 2-tailed unpaired Student’s *t* test; **P < 0.01.

Ten patients with VKH treated by TOFA who had not previously received systemic therapy and were glucocorticoid intolerant were included in this study. We found that a dose (5 mg, twice daily) of TOFA combined with a peribulbar injection of triamcinolone acetonide was safe and not associated with severe adverse events. According to the optical coherence tomography (OCT) examination, the inflammation gradually resolved with enhanced best-corrected visual acuity (BCVA) after 3 months of treatment (Figure 1, A and B, and Supplemental Tables 1 and 2; supplemental material available online with this article; https://doi.org/10.1172/jci.insight.162335DS1). TOFA treatment sufficiently controlled VKH symptoms in a similar manner when compared with 18 individuals treated with conventional therapy (systemic glucocorticoids and peribulbar injection of triamcinolone acetonide) (Figure 1A).

The differentiation of pathological TC subsets, Th1 and Th17, is mediated by inflammatory cytokines that signal through JAK/STAT pathways. We found that TOFA treatment reduced the percentages of IFN-γ^+^ Th1 and IL-17A^+^ Th17 (Figure 1, C and D). Additionally, TOFA treatment increased FOXP3 expression in CD4^+^ TCs (Figure 1E), indicating that TOFA treatment reversed Th17/Treg imbalance. We also investigated the ability of TOFA to inhibit cytokine signaling in TCs. As shown in Supplemental Figure 1, A and B, IL-2 induced STAT1 and STAT3 phosphorylation, and TOFA inhibited both events. TOFA cytotoxicity was assessed using a CCK8 assay, and we found no substantial cytotoxicity of the drug when concentrations less than 10 μM were used (Supplemental Figure 1C). Our results showed favorable antiinflammatory activity of TOFA in vitro.

### Strategy for single-cell immunophenotyping on peripheral blood from healthy individuals and patients with VKH.

To characterize the circulating immune modulation and explore the mechanisms of TOFA in VKH, we conducted single-cell tests using blood samples collected from healthy individuals (HC, *n* = 5) and patients before (VKH, *n* = 5) and after TOFA treatment (TOFA, *n* = 5), and wielded CyTOF and scRNA-Seq analysis (Supplemental Tables 3 and 4). CyTOF nodes were annotated into 3 main immune types (natural killer and TCs [NK&TCs], BCs, and myeloid cells [MYEs]) (Supplemental Figure 1, D and E). These populations were reorganized into 23 subsets (Supplemental Figure 1F). With scRNA-Seq, unsupervised analysis authenticated megakaryocytes, CD34^+^ cells, and 3 major immune populations (NK&TCs, BCs, and MYEs) according to the expression of canonical markers (Supplemental Figure 2A). Consistent with CyTOF data, we reclassified these into 25 transcriptionally conventional subsets (Supplemental Figure 2, B–D). The clustering analysis was based on published studies (23, 24). To unveil the transcriptional events implicated in VKH and TOFA treatment, differentially expressed genes (DEGs) between VKH and HC and between TOFA and VKH were identified as “VKH-DEGs” and “TOFA-DEGs,” respectively.

### Reconstituting the circulating cellular ecosystem of VKH.

**Figure 2:**
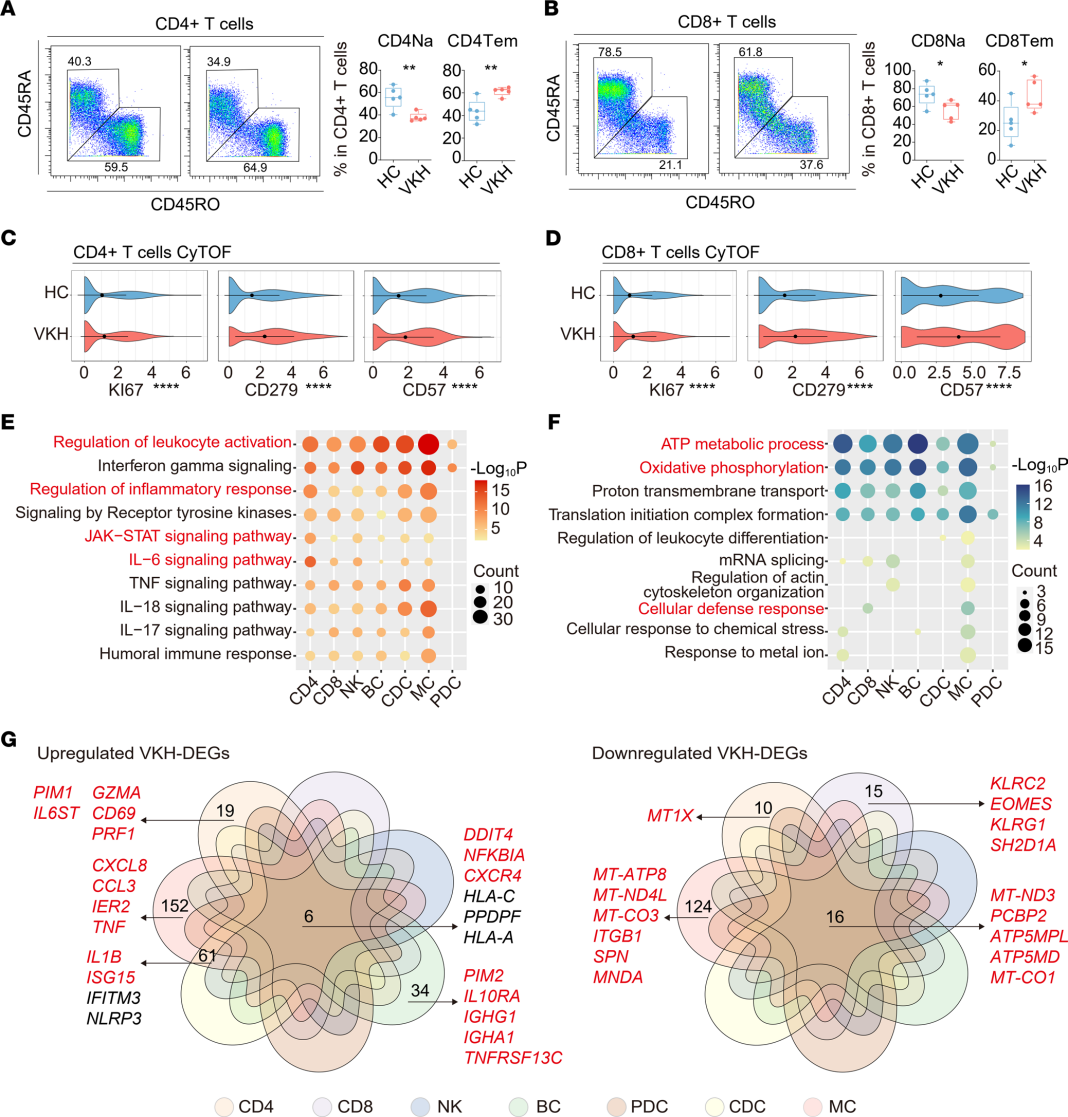
Reconstitution of the circulating cellular ecosystem by VKH. (**A**) The flow cytometry histograms showing the expression of CD45RA and CD45RO on CD4^+^ TCs, as well as the percentage of CD4^+^ Na and CD4^+^ Tem in CD4^+^ TCs between HC and VKH groups (*n* = 5/group). (**B**) The flow cytometry histograms showing the expression of CD45RA and CD45RO on CD8^+^ TCs and the percentage of CD8^+^ Na and CD8^+^ Tem in CD8^+^ TCs between HC and VKH groups (*n* = 5/group). Violin plot showing the expression of Ki67, CD279, and CD57 in CD4^+^ (**C**) and CD8^+^ (**D**) TCs between HC and VKH groups in CyTOF. Representative gene ontology (GO) biological process and pathways enriched in upregulated (**E**) and downregulated (**F**) VKH-DEGs among immune subsets. (**G**) Venn diagram showing the interactions of upregulated (left) and downregulated (right) VKH-DEGs among immune subsets. Significance in **A** and **B** was calculated using 2-tailed unpaired Student’s *t* test; *P < 0.05, **P < 0.01, ****P < 0.0001.

Based on the subpopulation results, we classified the 5 immune cell populations and projected a t-distributed stochastic neighbor embedding (t-SNE) diagram using CyTOF. By comparing the ratio between the HC and VKH groups, we noticed an increase in the percentage of monocytes (MCs) (Supplemental Figure 3, A and B), mainly due to the increase of CD14^+^ classical MCs (CMCs) (Supplemental Figure 3C). Subsequently, we found that VKH altered the composition and functional protein expression of TCs. Using CyTOF, we validated the downregulation in naive TCs (Na) and the increase in effector memory TCs (Tem) among both CD4^+^ and CD8^+^ TCs (Figure 2, A and B). The naive markers (CD45RA and LEF1) were decreased, and the memory and effector marker CD45RO was increased, with diminished CCR4, a Treg marker (Supplemental Figure 3D). Moreover, VKH increased several effector markers, including the proliferating marker Ki67, exhausted marker CD279, cytotoxic marker CD57, and T-bet (Figure 2, C and D, and Supplemental Figure 3D). Together, the results validated the complex cell dynamics in circulation altered by VKH, using CyTOF analysis.

Next, we explored transcriptional patterns of VKH. According to the number of VKH-DEGs, MCs were the subset most affected by VKH (Supplemental Figure 3E). We investigated the biological impact of the upregulated and downregulated VKH-DEGs via gene ontology (GO) and pathway analysis. The upregulated DEGs in CD4^+^ TCs were enriched in the IL-6 and JAK/STAT pathways (Figure 2E). VKH also upregulated multiple pathways involved in leukocyte activation and cytokine signaling, especially in MCs. In addition, the commonly downregulated genes were enriched in oxidative phosphorylation and ATP metabolism (Figure 2F). We then generated Venn diagrams to identify the interactions of VKH-DEGs in the 7 subsets (Figure 2G) and demonstrated that the commonly upregulated DEGs were *DDIT4*, *NFKBIA*, and *CXCR4*. DEGs related to inflammatory responses, including *CXCL8*, *CCL3*, *IER2*, and *TNF*, were only upregulated in MCs. Additionally, *IL1B* and *ISG15* were elevated in MCs and conventional DCs (CDCs). DEGs were only elevated in CD4^+^ TCs, including genes involved in JAK/STAT signaling (*PIM1*, *IL6ST*) and TCs’ activation (*GZMA*, *CD69*, and *PRF1*). Similarly, the upregulated VKH-DEGs specific to BCs were related to JAK/STAT signaling (*PIM2* and *IL10RA*) and BCs’ activation (*IGHG1*, *IGHA1*, and *TNFRSF13C*). In addition, the downregulation of the cellular defense response in CD8^+^ TCs was driven by *KLRC2*, *EOMES*, *KLRG1*, and *SH2D1A* (Figure 2G).

**Figure 3:**
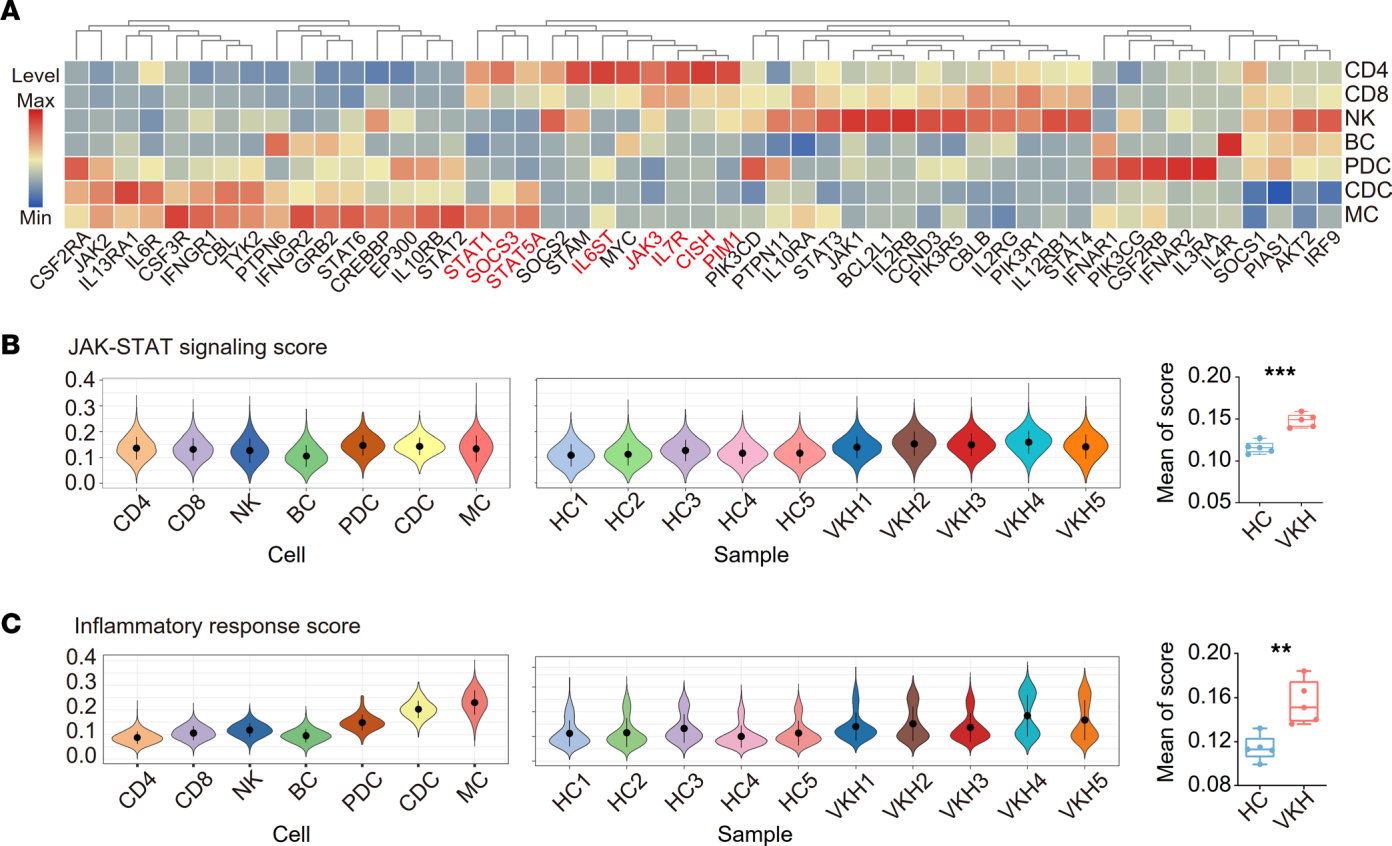
VKH induces the activation of JAK/STAT signaling and inflammatory response. (**A**) The heatmap showing the levels of genes associated with JAK/STAT signaling among immune subsets. (**B**) Violin plot showing the JAK/STAT signaling scores among different cells and samples. The scores were averaged between HC and VKH groups (*n* = 5/group). (**C**) Violin plot showing the inflammatory response scores among different cells and samples. The scores were averaged between HC and VKH groups (*n* = 5/group). Significance in **B** and **C** was calculated using 2-tailed unpaired Student’s *t* test; **P < 0.01, ***P < 0.001.

According to the upregulation of JAK/STAT pathways in the VKH group, we explored the levels of genes associated with JAK/STAT signaling in different cells (Figure 3A). We found that most genes were highly expressed in MCs. The genes that were highly expressed in CD4^+^ TCs included *IL6ST*, *JAK3*, *IL7R*, and *PIM1* and several others related to transcription factors (*STAT1*, *SOCS3*, and *STAT5A*) (Figure 3A). The biological scores were calculated to appraise the extent of the manifestation enhanced by VKH. The JAK/STAT signaling score was similar among subsets and was upregulated in patients with VKH compared with that in healthy individuals (Figure 3B). Furthermore, we found that VKH upregulated the inflammatory response score, with MCs showing the highest inflammatory response scores (Figure 3C). Finally, we averaged the scores and found that the mean scores in the TOFA group were significantly higher than those in the control group (Figure 3, B and C). In summary, we constructed a blood immune cell map of patients with VKH using CyTOF and scRNA-Seq. The decrease in the naive subset and increase in memory and effector subsets, cell activation upregulation, and the JAK/STAT pathway demonstrate that VKH induces cell-specific inflammatory states.

### Remodeling of the compartment and function of blood NKs and TCs by TOFA.

**Figure 4:**
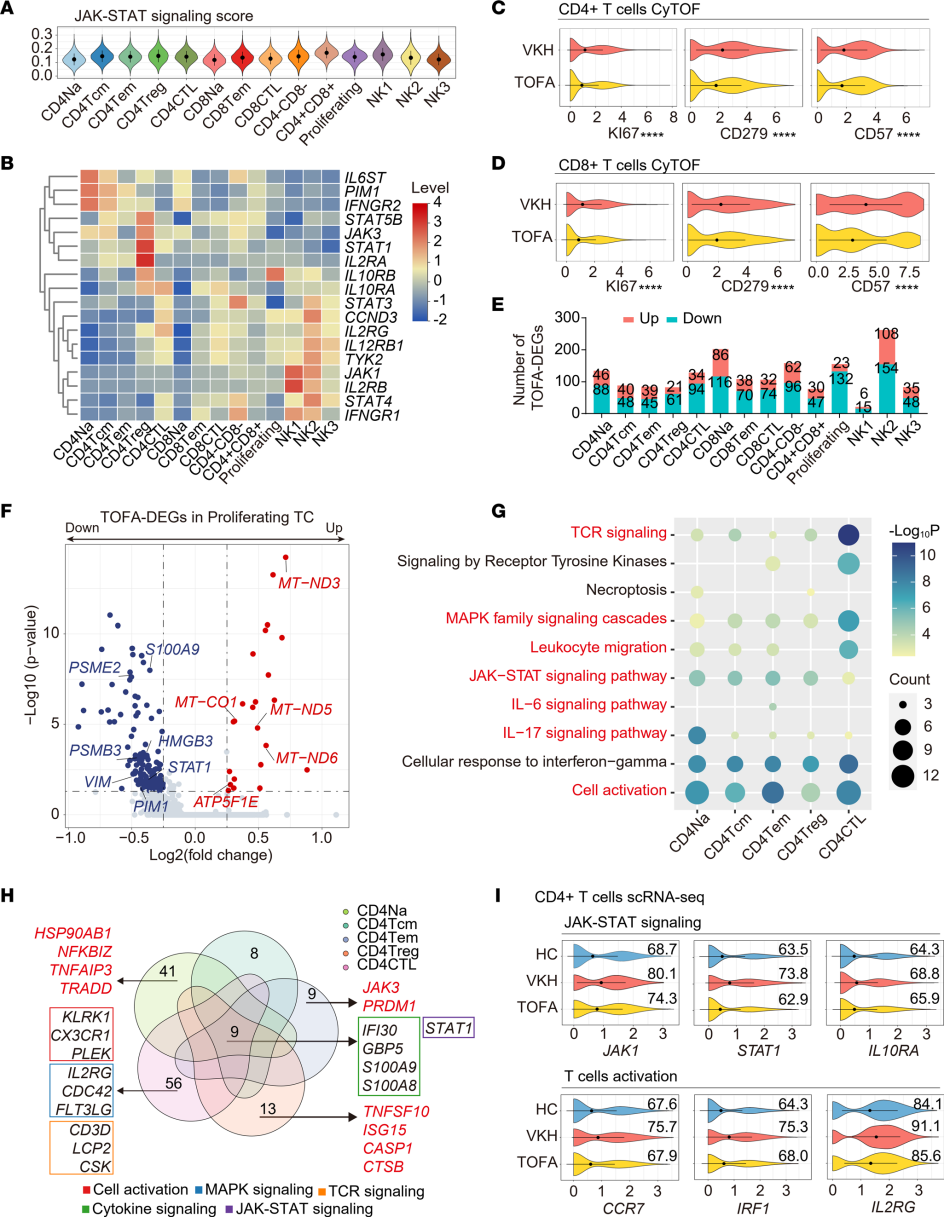
TOFA treatment downregulates cell activation of NKs and TCs. (**A**) Violin plot showing the JAK/STAT signaling score among NK&T subsets. (**B**) The heatmap showing the levels of genes associated with JAK/STAT signaling among NK&TC subsets. Violin plot showing the expression of Ki67, CD279, and CD57 in CD4^+^ (**C**) and CD8^+^ (**D**) TCs between HC and VKH groups in CyTOF. (**E**) The up- and downregulated TOFA-DEGs among NK&TC subsets. (**F**) Volcano plot showing TOFA-DEGs of proliferating TCs. (**G**) Representative GO biological processes and pathways enriched in downregulated TOFA-DEGs among CD4^+^ TC subsets. (**H**) Venn diagram shows the interactions of downregulated TOFA-DEGs among CD4^+^ TCs subsets. (**I**) Violin plot showing the expression of *JAK1*, *STAT1*, *IL10RA*, *CCR7*, *IRF1*, and *IL2RG* in CD4^+^ TCs among HC, VKH, and TOFA groups in scRNA-Seq.

Enhanced lymphocyte function is important for autoimmune diseases. As shown in the violin plots, the JAK/STAT signaling score was lowest in naive subsets and higher in highly differentiated memory and effector subsets (Figure 4A). By exploring the expression heterogeneity of JAK-related genes, we found that some genes (*PIM1* and *IL6ST*) were highly expressed in naive subsets, whereas most genes were highly expressed in memory and effector subsets (Figure 4B). The polarized and activated states of CD4^+^ and CD8^+^ TCs in patients with VKH indicated the importance of JAK/STAT signaling in the disease course. Notably, CyTOF validated that the increase of CD45RO, as well as the decrease of CD45RA, LEF1, and CCR4, were rescued by TOFA treatment (Supplemental Figure 4, A and B). In addition, TOFA inhibited the expression of markers related to TCs’ polarization, such as CD279, CD57, and T-bet. Accordingly, TOFA treatment led to a decrease in cytotoxic TCs and an increase in naive CD4^+^ and CD8^+^ TCs (Supplemental Figure 4, C and D). Moreover, Ki67 was downregulated after treatment (Figure 4, C and D). TOFA treatment reversed VKH-induced cell polarization and activation of CD4^+^ and CD8^+^ TCs.

Next, we explored the transcriptional patterns of TOFA in NK and TC subsets. According to the downregulated TOFA-DEG number, the subset most altered by TOFA was the CD56^mid^ CD57^–^ NK (NK2), followed by proliferating TCs (Figure 4E). Unsupervised analysis revealed that TOFA therapy decreased the expression of genes related to inflammation and JAK/STAT pathway (*STAT1*, *PIM1*) and upregulated the level of genes associated with oxidative phosphorylation in the proliferating subset (Figure 4F). In addition, CD8^+^ TCs and NKs were most affected by TOFA treatment (Figure 4E). Using CyTOF, we validated that the levels of cytotoxic factors were decreased by TOFA, such as CCL5 and granzyme K (GZMK) in NKs and CCL5 and GZMB in CD8^+^ TCs (Supplemental Figure 4, E and F). We further investigated the transcriptional characteristics modified by TOFA by performing functional analysis. We found that TOFA treatment downregulated various cytotoxicity-related pathways, including cytokine signaling and cell activation and differentiation, especially in NK2, CD8^+^ Tem, and CD8^+^ cytotoxic T lymphocytes (CTLs) (Supplemental Figure 4G).

The inhibitory effects of TOFA on pathological CD4^+^ subsets (Th1 and Th17) in vitro are shown above (Figure 1, C and D). Therefore, we explored the transcriptional patterns of TOFA on CD4^+^ TCs. Functional analyses revealed that the VKH-induced overrepresentation of JAK/STAT signaling in CD4^+^ TCs was reduced by TOFA treatment (Figure 4G). In the CD4^+^ CTL subset, TOFA downregulated leukocyte migration, as well as TCR and MAPK signaling. In addition, cell activation and IL-6 signaling were downregulated, especially in the CD4^+^ Tem subset, whereas IL-17 signaling was downregulated, especially in the CD4^+^ Na subset (Figure 4G). Using Venn plots to assess the subset-specific signatures of TOFA, we found that all CD4^+^ subsets showed decreased expression of genes related to cytokine and JAK/STAT signaling (*STAT1*) (Figure 4H). Downregulation of the IL-17 signaling pathway in CD4^+^ Na was attributed to *HSP90AB1*, *NFKBIZ*, *TNFAIP3*, and *TRADD*, and that of the IL-6 signaling pathway in CD4^+^ Tem was driven by *JAK3* and *PRDM1* (Figure 4, G and H). Notably, several inflammation-related genes (*TNFSF10*, *ISG15*, and *CASP1*) were inhibited by TOFA in CD4^+^ Tregs, indicating that TOFA had a therapeutic effect in reversing inflammatory damage in Tregs. Next, we performed an integrative comparative analysis of the VKH- and TOFA-DEGs. We found that genes related to JAK/STAT signaling (*JAK1*, *STAT1*, and *IL10RA*) and TCs’ activation (*CCR7*, *IRF1*, and *IL2RG*) were increased in VKH and decreased after TOFA treatment (Figure 4I).

Collectively, TOFA remodeled the compartment and function of blood NKs and TCs and decreased markers and processes related to cell polarization, cytotoxic functions, and autoimmune signaling pathways.

### TOFA rescuing of VKH-induced IFN signaling and activation of BCs.

**Figure 5:**
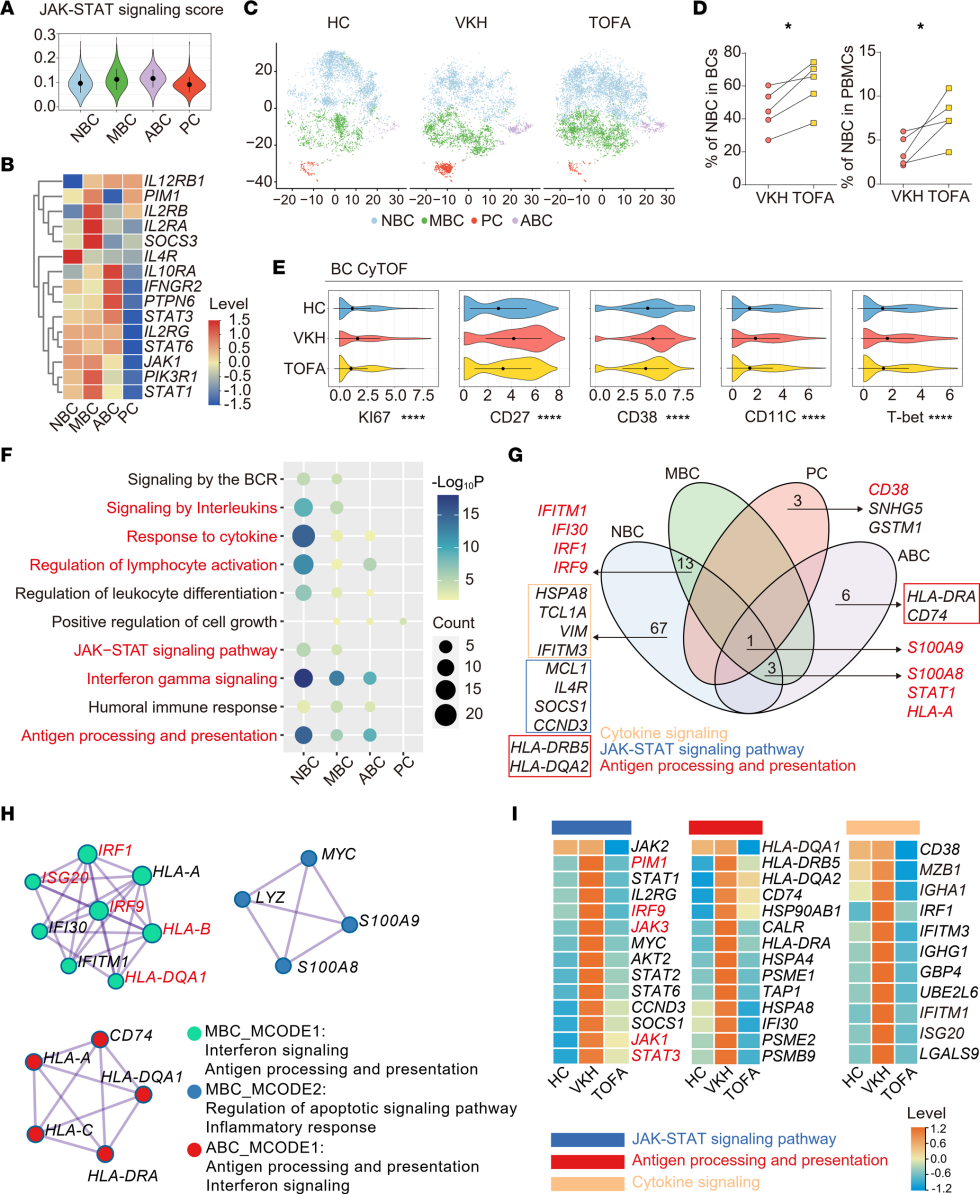
TOFA treatment downregulates cell activation of BCs. (**A**) Violin plot showing the JAK/STAT signaling score among BC subsets. (**B**) The heatmap showing the levels of genes associated with JAK/STAT signaling among BC subsets. (**C**) t-SNE plot of BC subsets among 3 groups. (**D**) The percentage of naive B cells in BCs or PBMCs between HC and VKH groups (*n* = 5/group). (**E**) Violin plot showing the expression of Ki67, CD27, CD38, CA11C, and T-bet in BCs among 3 groups in CyTOF. (**F**) Representative GO biological processes and pathways enriched in downregulated TOFA-DEGs among BC subsets. (**G**) Venn diagram shows the interactions of downregulated TOFA-DEGs among BC subsets. (**H**) The gene network showing protein-protein interaction (PPI) analysis of downregulated TOFA-DEGs in the memory B cell subset. (**I**) The heatmap showing the levels of genes associated with specific pathways in BCs among HC, VKH, and TOFA groups. Significance in **D** was calculated using 2-tailed paired Student’s *t* test; *P < 0.05, ****P < 0.0001. ABC, autoimmune-associated B cell; PC, plasma cell.

Among the BCs, we found that the JAK/STAT signaling score was lowest in naive subsets and highest in highly differentiated subsets (Figure 5A) in accordance with the expression heterogeneity of JAK-related genes (Figure 5B). We found that the memory subset was increased in the VKH group and downregulated in the TOFA counterpart, and the naive subset showed the opposite transition, indicating the TOFA reversal effect (Figure 5, C and D, and Supplemental Figure 5, A and B). Similarly, using CyTOF, we validated that TOFA treatment reduced the expression of Ki67 and the activation markers (CD27 and CD38) and downregulated the autoimmune-related BC markers, T-bet and CD11C (Figure 5E).

Next, we assessed the TOFA-modulated transcriptional signatures of BC subsets. TOFA treatment decreased the expression of genes related to inflammation and BC activation and upregulated the level of genes associated with oxidative phosphorylation (Supplemental Figure 5C). In addition, the downregulated TOFA-DEGs in naive BCs were enriched in the regulation of lymphocyte activation, antigen processing, and presentation and the IFN-γ signaling pathway (Figure 5F). Using Venn plots to appraise the subtype-specific characteristics, we found that all BC subsets showed decreased *S100A9* expression. Besides, we ascertained subtype-specific profiles of TOFA, incorporating IFN-related genes (*IFITM1*, *IFI30*, *IRF1*, and *IRF9*) in naive and memory BCs, BC differentiation-related genes (*CD38*) in plasma BCs, *HLA-DRA* and *CD74* in autoimmune-related BCs, and inflammation-related genes (*S100A8*, *STAT1*, and *HLA-A*) in 3 BC subsets (Figure 5G). In addition, downregulation of biological processes indicated that TOFA treatment had extensive modulatory functions in naive BCs (Figure 5, F and G).

Protein-protein interactions (PPIs) are a complex web of functional associations between biomolecules (25). Potential protein complexes can be identified computationally from PPI networks by applying molecular complex detection (MCODE) method, which flags groups of entities interacting in certain patterns (26). Therefore, we performed PPI analysis on DEGs of BC subsets to explore subset-specific gene networks. The top 2 signaling pathways corresponding to each MCODE were shown. In the memory BCs, 2 types of MCODEs were identified, with the most enriched being IFN signaling, antigen processing, and presentation processes (Figure 5H). Notably, *IRF9* had the strongest correlation with other genes, which is vital in BCs’ activation and inflammatory response mediated by IFN-β and downstream JAK/STAT pathway (27, 28) (Figure 5H). For naive BCs, the most enriched MCODEs were in IFN and cytokine signaling (Supplemental Figure 5D). Additionally, we targeted genes related to 3 specific pathways among groups. TOFA treatment rescued the VKH-induced upregulation of genes related to the JAK/STAT pathway (*IRF9*, *JAK1*, *STAT3*, and *PIM1*), antigen processing and presentation, and cytokine signaling (Figure 5I). Taken together, these findings highlight the rescuing effects of TOFA treatment on BCs’ polarization and activation, as well as the signaling pathways related to inflammatory and autoimmune disorders.

### Effect of TOFA treatment on inflammation- and JAK-associated CMC subsets.

**Figure 6:**
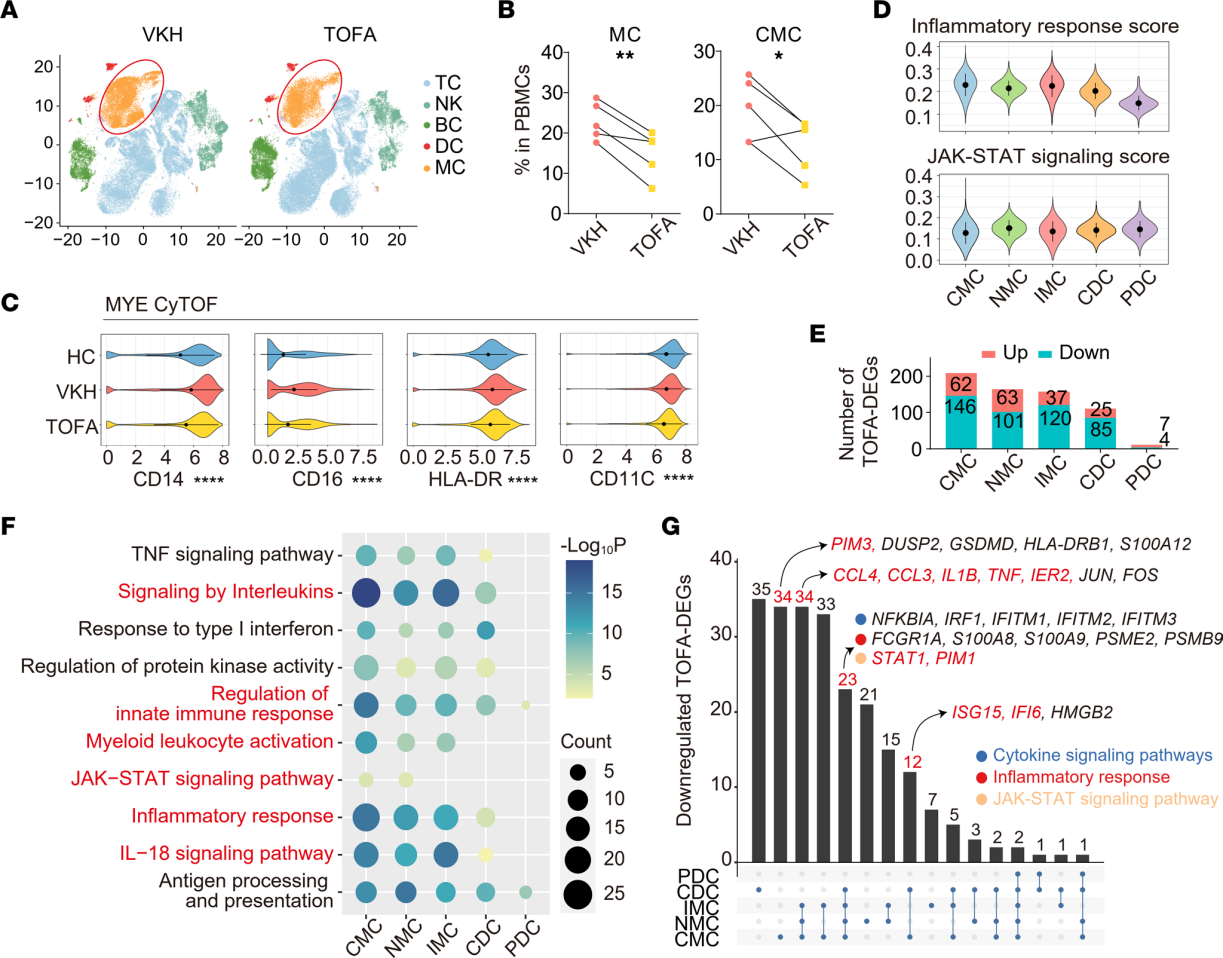
TOFA treatment downregulates cell activation of MYEs. (**A**) t-SNE plot of immune cells in VKH and TOFA groups. (**B**) The percentage of MCs and CMCs in PBMCs between HC and VKH groups (*n* = 5/group). (**C**) Violin plot showing the expression of CD14, CD16, HLA-DR, and CD11C in MYEs among HC, VKH, and TOFA groups in CyTOF. (**D**) Violin plot showing the inflammatory response score and JAK/STAT signaling score among MYE subsets. (**E**) The up- and downregulated TOFA-DEGs in MYE subsets. (**F**) Representative GO biological process and pathways enriched in downregulated DEGs among MYE subsets. (**G**) UpSet plot showing the integrated comparative analysis of downregulated TOFA-DEGs in MYE subsets. Significance in **B** was calculated using 2-tailed paired Student’s *t* test; *P < 0.05, **P < 0.01, ****P < 0.0001. NMC, nonclassical monocyte; IMC, intermediate monocyte; PDC, plasmacytoid dendritic cell.

Analysis of patients’ data indicated that patients with VKH had an increase of MCs and CMCs, suggesting the possible role of MCs in VKH disease. Notably, TOFA treatment decreased the abnormal immune cell changes (Figure 6, A and B). Next, we compared the expression of functional markers in MYEs among 3 groups. We demonstrated that the upregulated markers in VKH were decreased after TOFA treatment, including CD14, CD16, and markers related to antigen presentation (HLA-DR and CD11C) (Figure 6C).

Thereafter, we elucidated transcriptional changes in MYEs. The CMC subset showed the highest score in inflammatory response and had the widest span in the JAK/STAT signaling score, as indicated in the violin plots (Figure 6D). By exploring the expression heterogeneity across subpopulations, we found that *IFNAR1*, *IFNAR2*, and *JAK1* were highly expressed in the plasmacytoid DCs, whereas some genes (*JAK3*, *STAT3*, *IL6R*, *IL6ST*, *PIM1*, and *CSF3R*) were highly expressed in CMCs (Supplemental Figure 6A). Consistent with patients’ data, MCs (especially CMCs) were the subset most influenced by TOFA (Figure 6E). Accordingly, we found that TOFA treatment downregulated cytokine signaling, myeloid leukocyte activation, and JAK/STAT signaling, all of which contribute to inflammatory disorders. The modulatory effects were especially prominent in CMCs (Figure 6F). Next, we separated the TOFA-DEGs on each MYE subset (Figure 6G). The expression of inflammation-related genes decreased in all MC subsets, including *CCL3*, *CCL4*, *IL1B*, *TNF*, and *IER2*. In addition, subtype-specific patterns were also identified, including JAK-related genes (*STAT1* and *PIM1*) in CDCs and all MC subsets, *PIM3* in CMCs, and *ISG15* and *IFI6* in CMCs and CDCs (Figure 6G).

**Figure 7:**
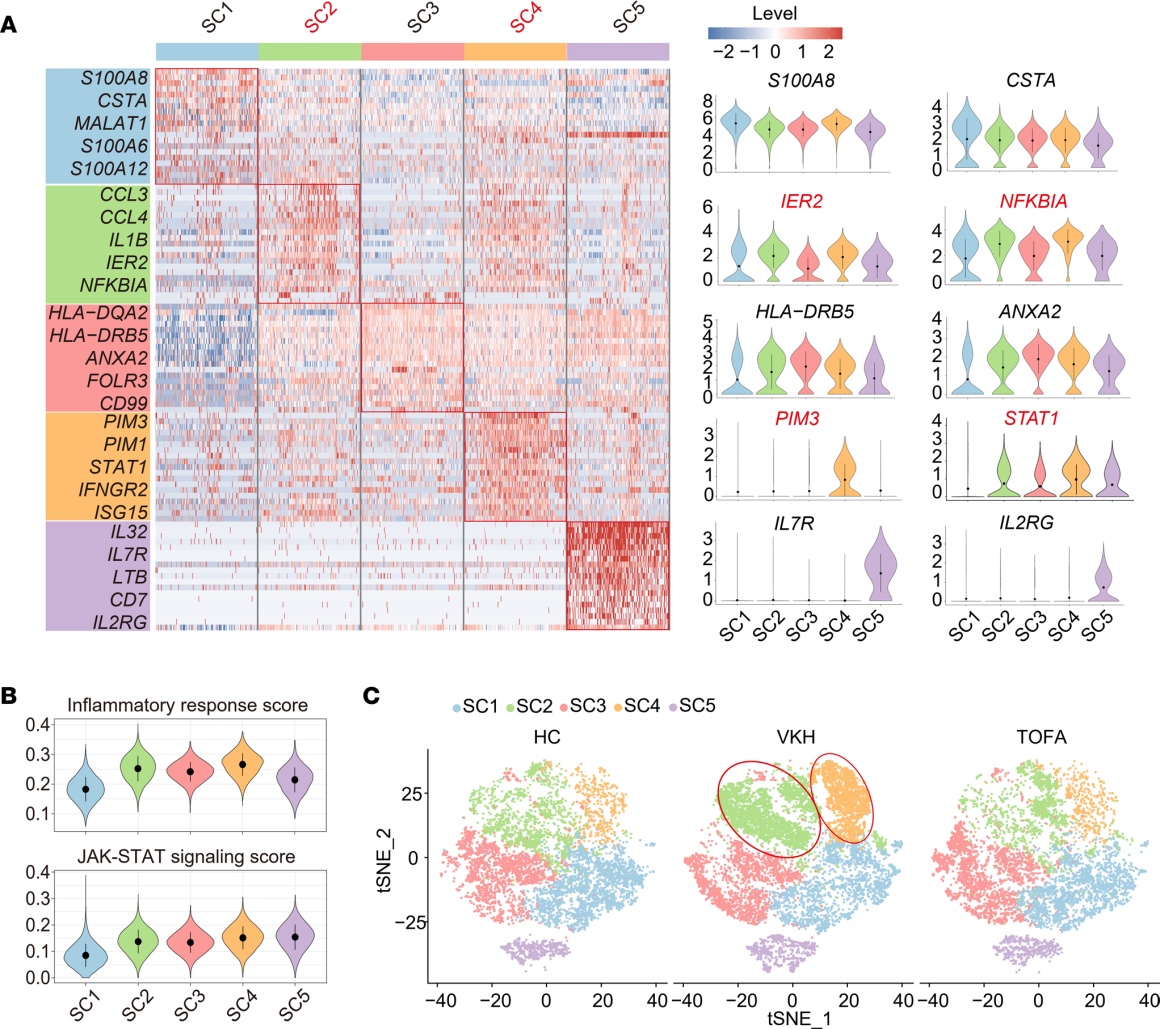
TOFA treatment downregulates cell activation of CMCs. (**A**) The heatmap showing the scaled expression of discriminative gene sets of the CMC SCs (left) and violin plot showing the expression of specific genes among CMC SCs (right). (**B**) Violin plot showing the inflammatory response score and JAK/STAT signaling score among CMC SCs. (**C**) t-SNE plot of CMC SCs among 3 groups.

It is known that CMCs belong to the first line of immune defense cells, are important antigen-presenting cells recruited to lesion sites during infection or sterile injury, and contribute to autoimmune disease development (7, 29–31). However, disease-specific MC populations with unique functions and signatures remain largely unknown. Therefore, we reclustered the CMCs into 5 subclusters (SC1–5) (Figure 7A). Both SC2 and SC4 had high expression of several inflammatory genes (*IER2* and *NFKBIA*) and were characterized by the highest inflammatory response score (Figure 7, A and B). In addition, both SC4 and SC5 had higher JAK/STAT signaling scores than other SCs, with *PIM3*, *PIM1*, and *STAT1* and cytokine receptors (*IL7R* and *IL2RG*), respectively (Figure 7, A and B). Notably, the 2 VKH-expanded subclusters, SC2 and SC4, which were related to inflammatory response and JAK/STAT signaling, were diminished by TOFA treatment (Figure 7C and Supplemental Figure 6B). These results suggest that inflammation- and JAK-related CMC subgroups are highly associated with VKH disease and TOFA treatment. Seven MCODEs were identified from the downregulated TOFA-DEGs of CMCs, all of which were related to inflammatory response, myeloid leukocyte activation, and JAK/STAT signaling (Supplemental Figure 6C). Integrative comparative analysis also indicated that the expression of related genes was increased in the VKH group and rescued by TOFA treatment (Supplemental Figure 6D).

### Demonstration of TOFA rescuing effect via predisposing genes and intercellular communication profiles of VKH.

**Figure 8:**
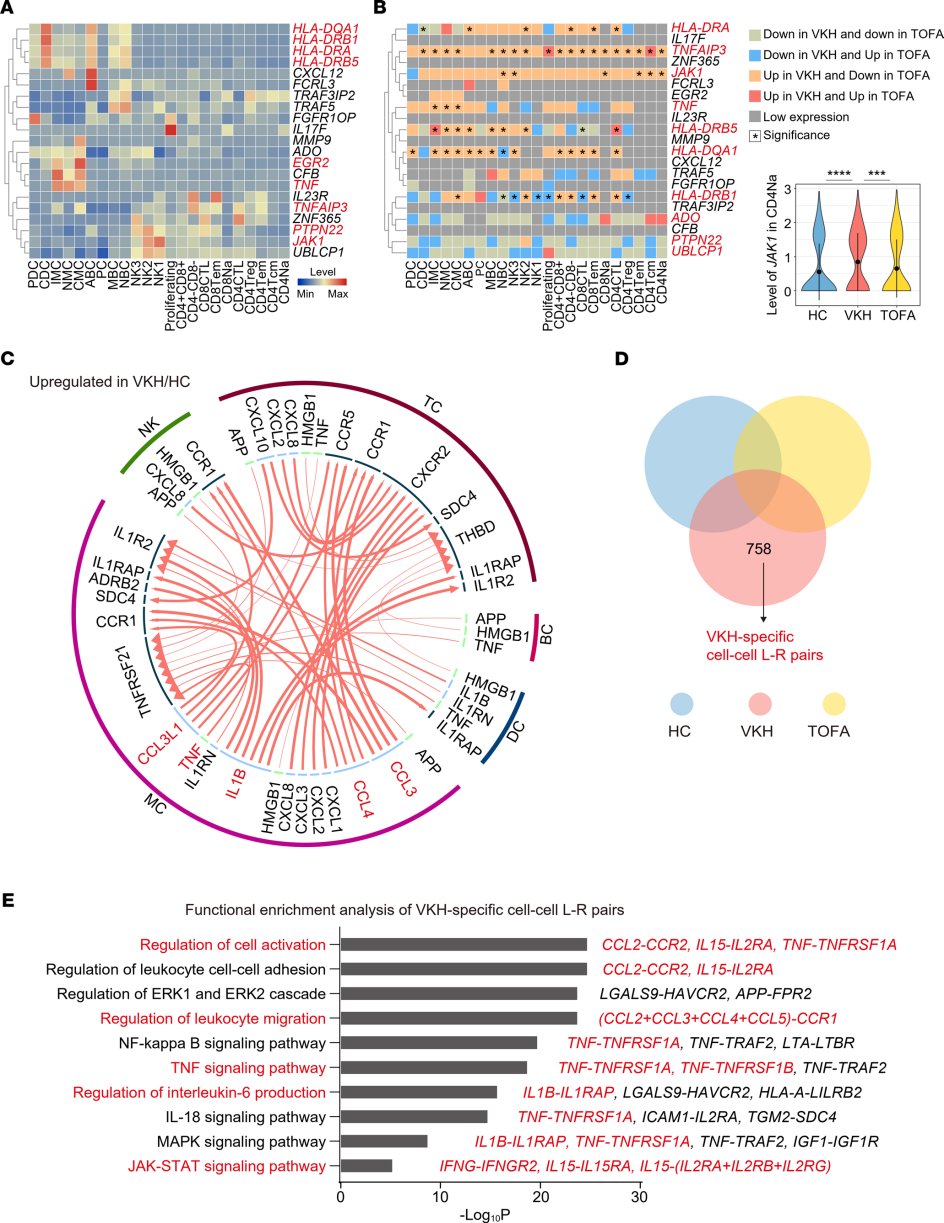
TOFA treatment rescues the susceptibility genes and cell-cell interaction influenced by VKH. (**A**) The heatmap representing scaled expression levels of VKH susceptibility genes (*n* = 21) across the 23 immune subclusters. (**B**) The heatmap representing the variation trends of VKH susceptibility genes across the 23 immune subclusters. The expression differences of related genes influenced by VKH and TOFA treatment are indicated by color. The asterisk symbol in the heatmap indicates statistical difference in gene expression between VKH and HC or between TOFA and VKH. The violin plot shows the relative level of JAK1 in CD4^+^ Na subset among the 3 groups. Significance was calculated using 2-sided Wilcoxon test; ***P < 0.001, ****P < 0.0001. (**C**) Circle plot showing the upregulated L-R pairs in VKH/HC comparison. (**D**) Venn diagram showing the analysis of VKH-specific cell-cell L-R pairs. (**E**) Representative GO biological process and pathways enriched in VKH-specific cell-cell L-R pairs.

Genetic factors have been shown to be implicated in the development of VKH (32). The identification of susceptibility genes for VKH can be used as a reliable basis to recognize similarities as well as differences among VKH and other types of uveitis. As such, we first addressed the cellular distribution of VKH susceptibility genes across the PBMC subclusters by referring to a study employing single nucleotide polymorphisms associated with VKH (33). These are some genes that encode proteins involved in multifarious pathways connected to VKH pathogenesis, including the antigen presentation processes (*HLA-DQA1*, *HLA-DRB1*, *HLA-DRA*, and *HLA-DRB5*), inflammatory activation (*CXCL12*, *FCRL3*, *MMP9*, and *TRAF5*), cytokine signaling pathways (*TNF*, *IL23R*, and *JAK1*), as well as the regulation of immune response (*EGR2* and *PTPN22*). In this study, we first analyzed the expression of the genes in blood immune cells (Figure 8A) and found that *EGR2*, *TNF*, and genes related to antigen presentation were mainly expressed in MYEs, while *JAK1*, *TNFAIP3*, and *PTPN22* were primarily expressed in lymphocytes (Figure 8A). We then illustrated how the transcriptional levels of these genes were influenced by VKH or TOFA treatment. We found that *TNF* was increased in MYEs of the VKH group and decreased after TOFA therapy (Figure 8B). Interestingly, *JAK1* showed similar variation trends in lymphocytes, as well as *HLA-DQA1* and *TNFAIP3* in almost all types of immune cells. Detailed information combining the published risk loci of VKH susceptibility genes (33) with our results are summarized in Supplemental Table 5. In addition, TNFAIP3 has been involved in the negative feedback regulation of the inflammatory NF-κB pathway, and its expression is decreased in ocular Behcet’s disease, which is another type of uveitis (34). Employing real-time quantitative PCR, we found that *TNFAIP3* gene expression level was increased in VKH relative to other types of uveitis. Similar trends were noted for other gene families involved in the NF-κB signaling pathway, *NOD1* and *NOD2* (Supplemental Figure 7A); this was in agreement with other studies (35). These data, therefore, provided an opportunity to shed more light on the potential functional mechanisms for genetic risk loci in VKH disease, particularly the cell-specific increase of VKH susceptibility genes, which can be partly suppressed by TOFA treatment.

Furthermore, recent advances in biotechnology provided opportunities to define function- or character-specific cell-cell communications (36). Although immune rescue by TOFA treatment was emphasized in the above results, the specific TOFA-modulated intercellular interactions in the human circulating immune system have not been investigated to our knowledge. To identify the intercellular interactions modified by TOFA, we first investigated the intercellular communication between VKH versus HC and TOFA versus VKH using the iTALK tool. We focused on the upregulated ligand-receptor pairs in VKH versus HC and the downregulated ligand-receptor (L-R) pairs in TOFA versus VKH. We found that VKH increased several interactions between MCs and other cells, which were mainly involved in the inflammatory activation and chemotaxis of MCs to other cells (Figure 8C). MC-secreted cytokines and chemokines encoded by *IL1B*, *TNF*, *CCL3*, and *CCL4*, all of which contribute to autoimmunity and inflammation, may stimulate other cell populations expressing their homologous receptors. TOFA treatment rescued the interactions involving these pairs. Notably, the therapeutic effects of TOFA were also reflected in the reduced communication between inflammatory genes (*TNF* and *IL6*) and the corresponding receptors between MCs and other cells (Supplemental Figure 7B). Additionally, we focused on the VKH-specific cell-cell L-R pairs, which were only detected in the VKH group (Figure 8D). Functional enrichment analysis indicated that these pairs were enriched in cell activation, leukocyte migration, and TNF and JAK/STAT pathways, which were driven by *TNF*- and *CCL*-related L-R pairs (Figure 8E).

**Figure 9:**
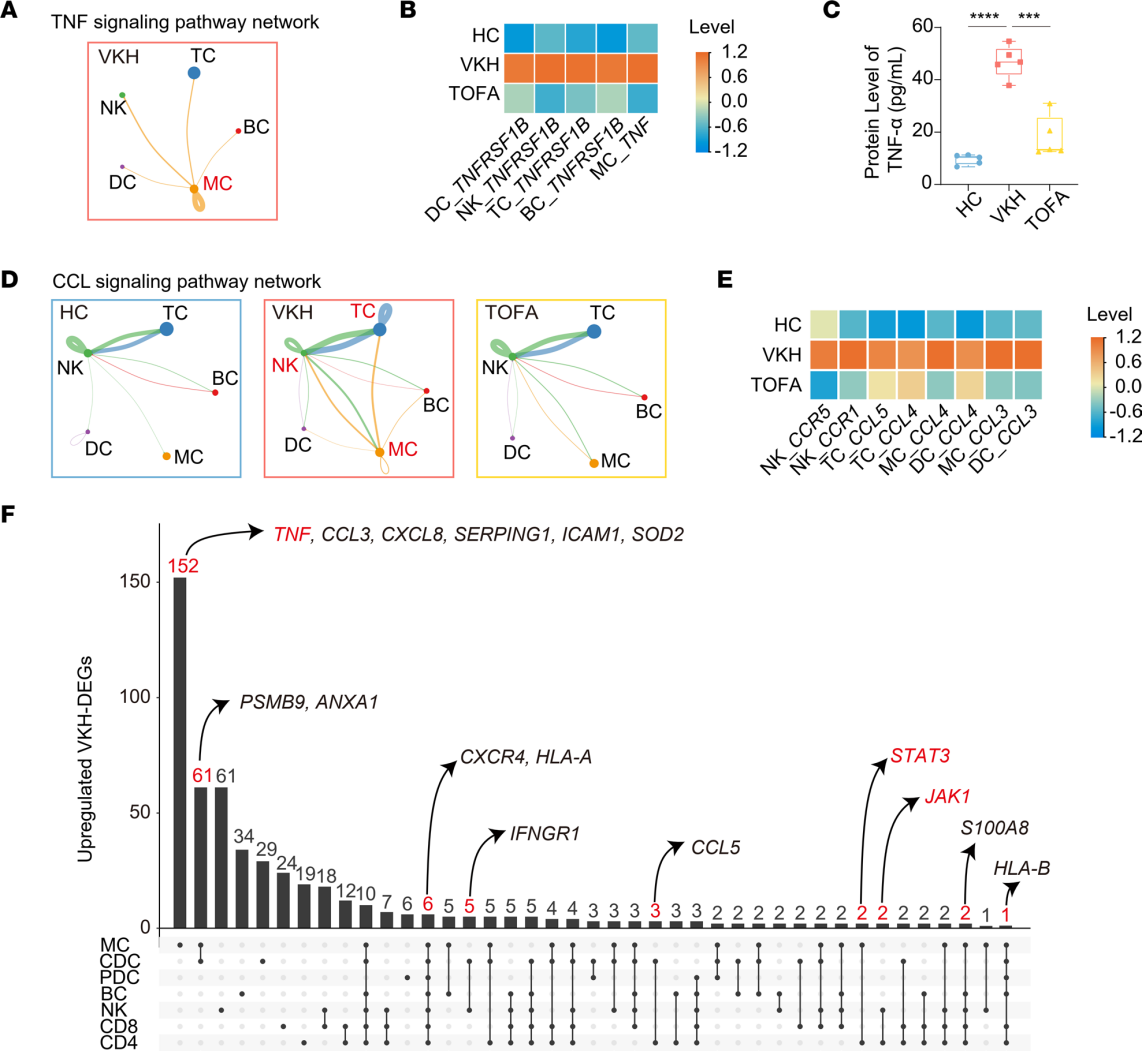
TOFA treatment reduces the inflammatory cytokine signaling influenced by VKH. (**A**) Circle plot showing TNF signaling pathway network only in VKH group. (**B**) The heatmap showing the levels of *TNF* and *TNFRSF1B* in immune cells among groups. (**C**) Protein level of TNF-α in serum of HC, VKH, and TOFA groups detected by ELISA. (**D**) Circle plot showing CCL signaling pathway network in HC, VKH, and TOFA groups. (**E**) The heatmap showing the levels of ligands and receptors related to CCL signaling in immune cells among groups. (**F**) UpSet plot showing the integrated comparative analysis of upregulated VKH-DEGs among immune subsets. The red numbers indicate the cellular distribution of 16 candidate genes among immune subsets, and the red text indicates the genes of interest involved in cytokine signaling pathways. Significance in **C** was calculated using 2-tailed unpaired Student’s *t* test; ***P < 0.001, ****P < 0.0001.

Afterward, we explored intercellular signaling using CellChat. This tool detected the TNF signaling pathway in the VKH group but not in the HC or TOFA counterparts, indicating the specific correlation between TNF signaling and VKH (Figure 9A). MCs were the primary TNF source, which acted in a paracrine manner toward other cells, with the *TNF*-*TNFRSF1B* L-R pair being the major driver (Figure 9A and Supplemental Figure 7C). The upregulation of *TNF* in MCs and *TNFRSF1B* in other cells was rescued by TOFA treatment (Figure 9B), which was confirmed by the protein level of TNF-α in the serum (Figure 9C). Moreover, we found that VKH upregulated the CCL signaling pathway mainly among MCs, TCs, and NKs, which were decreased in the TOFA group (Figure 9D). CCL signaling is involved in cell migration and activation, with the *CCL5*-*CCR1* L-R pair being a major signaling driver (Supplemental Figure 7D). The expression of related ligands and receptors in immune cells increased in the VKH group and decreased in the TOFA group (Figure 9E). We also found that several cytokine pathways related to JAK/STAT signaling were altered in specific cells. The IL-6 signaling pathway was driven by the intercellular *IL6*-(*IL6R*+*IL6ST*) pairs among cells (Supplemental Figure 7, E–G). Similar results were observed for the IFN-γ signaling pathway network between MCs and NKs (Supplemental Figure 7H). *IFNG*-(*IFNGR1*+*IFNGR2*) was the dominant contributor, and the upregulation of receptors in NKs and MCs in VKH was rescued by TOFA treatment (Supplemental Figure 7, I and J). These findings reveal that VKH-induced cell-cell pathways are implicated in inflammatory and autoimmune disorders and highlight the reversal effects of TOFA treatment.

In this study, we explored the cell-specific pathogenesis of VKH. The upregulation of JAK pathway, as well as cell activation and polarization, suggested the therapeutic effect of TOFA targeting JAK pathway in VKH. Therefore, we further mined our data to predict other potential therapeutic agents for VKH. Specifically, the upregulated genes in VKH were annotated by Metascape tool. Then, we screened the 32 uveitis-associated genes based on DisGeNET annotation. Finally, we screened the 16 uveitis-associated genes and their targeted drugs by referring to the DrugBank database (Supplemental Figure 7K). These genes were involved in immune cell chemotaxis (*CCL3*, *CCL4*, *CCL5*, *CXCL8*), TC activation and differentiation (*CXCR4*, *S100A8*), and cytokine pathways (*TNF*, *IFNGR1*, *JAK1*, *STAT3*). We next explored the cell-specific patterns and found that most of these candidate genes were upregulated in MCs of the VKH group, like *TNF* and *STAT3* (Figure 9F). The upregulation of these genes suggested the potential therapeutic effect of these novel targeted drugs for VKH treatment. For example, agents targeting TNF pathway (like adalimumab) have been very effective in the treatment of autoimmune diseases and have been the best-selling biologics on the market (37, 38). The increased expression of *TNF* in MCs from patients with VKH indicated that adalimumab may be an effective therapeutic drug for VKH disease. In addition, ENMD-1198 can directly inhibit STAT3, a key factor in Th17 cell differentiation and VKH development (39), suggesting potential applications in future studies. As such, our data may provide a basis for subsequent studies of these targeted drugs for VKH treatment.

## Discussion

Here, we first comprehensively demonstrated the implication of various immune parameters in VKH at proteomic and transcriptomic levels using CyTOF and scRNA-Seq. We systematically evaluated the effects of TOFA treatment on VKH according to cell type composition, subtype-specific gene expression, enriched pathways, and intercellular communication. The primary findings of the current study were as follows: 1) The immune dysregulations in VKH were characterized by TCs’ polarization from naive to effector, memory, and cytotoxic cells; increased inflammation- and JAK-associated MCs; and upregulated cell activation and cytokine and JAK/STAT signaling. 2) In vitro, TOFA reversed the Th17/Treg imbalance and inhibited IL-2–induced STAT1/3 phosphorylation. 3) Comparable improvement in clinical symptoms was noted between patients treated with TOFA and those receiving conventional therapy, suggesting that a systemic “glucocorticoid-free” strategy is feasible for treating VKH. 4) TOFA treatment reversed the VKH-induced downregulation of naive subsets and the increase in effector memory subsets among CD4^+^ and CD8^+^ TCs, as well as various pathways related to cytokine signaling, JAK/STAT pathway, and lymphocyte function. 5) The upregulation of antigen presentation and processing, inflammation activation, and overrepresentation of 2 inflammation- and JAK-related CMC subpopulations of the VKH group were rescued after TOFA treatment. 6) TOFA rescued VKH-induced upregulation of specific predisposing genes and cell-cell interactions implicated in the inflammatory response, cell migration, and cell activation.

VKH is a severe bilateral, granulomatous, intraocular, autoimmune, inflammatory disorder mediated by TCs, characterized by rapid onset and recurrent inflammation (5). Previous studies on VKH pathogenesis have mainly concentrated on the imbalance between pathological and regulatory TCs (40–42). However, some patients with little response to traditional therapy lack access to effective therapies. Therefore, a more specific understanding of VKH pathogenesis is necessary to facilitate the development of optimized therapies for patients with the disease. This study elucidated the VKH immune landscape with high resolution and precision using multimodal studies. Our data supported the documented increase of cell polarization in TCs and BCs (43, 44), characterized by increased proliferation, exhaustion, and cytotoxicity. Common VKH-upregulated pathways were involved in leukocyte activation, cytokine signaling, and the JAK/STAT pathway. Particularly, VKH commonly increased *DDIT4*, *NFKBIA*, and *CXCR4* expression levels. *CXCR4* has been shown to contribute to the pathogenesis of Lewy body dementia (45) and multiple sclerosis (46) by promoting TC recruitment and Th17/Treg imbalance. Combined with the results of VKH-induced cell-specific genes, such as the upregulation of *IGHG1*, *IGHA1*, and *TNFRSF13C* in BCs, VKH induced a proinflammatory and autoreactive status in immune system circulation. The higher expression of genes related to the JAK/STAT pathway and the polarized and activated states of CD4^+^ and CD8^+^ TCs indicated the importance of JAK/STAT signaling in VKH. In addition, VKH downregulated ATP metabolism and the Treg marker, CCR4, which is specifically expressed by effector Tregs and suppress aberrant immune responses against self-antigens (47). Furthermore, VKH was characterized by an increased proportion of MCs, especially CD14^+^ CMCs. The upregulated genes in CMCs were enriched in cell migration (*CCL3*, *CCL4*, and *CXCL8*) and inflammation (*TNF*, *IL1B*, and *ISG15*). As an important proinflammatory factor, the increased expression of TNF in MCs from patients with VKH could be a significant therapeutic target for the disease (38). Moreover, *ISG15* has been identified as a disease-specific gene that is preferentially enriched in the proinflammatory subset of patients with VKH (48). These results suggest the vital role of MCs in the development of VKH. We mapped the human circulating immune system of patients with VKH using multimodal tools, which was characterized by increased peripheral lymphocyte polarization, cell activation, JAK/STAT signaling, and inflammatory activation.

Early and aggressive systemic corticosteroids are still the mainstream therapy for VKH, notwithstanding immunosuppressive and biological agents, which are not as potent as instant interventions and which are becoming the norm in adjuvant therapy for corticosteroids (8). The ocular and systemic side effects of corticosteroids preclude prolonged use. Knowledge of inflammatory mediators in autoimmune disorders has facilitated the development of novel therapeutics that selectively target individual molecules. As a JAK1/3 inhibitor, TOFA was approved by the FDA for rheumatoid and psoriatic arthritis and ulcerative colitis (17, 18). The effectiveness identified in other autoimmune diseases and the suppression of TCs’ proliferation are practical foundations for using TOFA as a novel intervention in VKH. We present a comprehensive and integrated circulating immune cell landscape of patients with VKH characterized by upregulated cytokine and JAK/STAT pathways. Our study is the first to our knowledge to confirm the validity of TOFA treatment in patients with VKH, showing no discrepancy with conventional therapy. The OCT examination revealed that ocular inflammation was gradually resolved, with enhancement of BCVA, after TOFA treatment. More importantly, we confirmed a potentially novel and successful “zero systemic corticosteroids” approach, independent of systemic corticosteroids, controlling VKH with minimal side effects, which is a breakthrough in the context of VKH treatment. In addition, the study conducted by Zaka-ur-Rab et al. revealed that the peribulbar injection of triamcinolone acetonide at a dose of 40 mg would result in detectable triamcinolone acetonide in blood, with an obvious decrease at 48 hours after injection (49). In our study, TOFA was administered orally every day, in contrast to peribulbar injection of 20 mg triamcinolone acetonide once a month, indicating TOFA was the dominant treatment in our study. On the other hand, the blood triamcinolone acetonide level was much lower than the blood level of glucocorticoid that achieved antiinflammatory effects after conventional oral prednisolone administration (0.5 mg/kg), as well as the daily physiological dose in glucocorticoid replacement therapy. Moreover, the detectable triamcinolone acetonide level was also much lower than the effective concentration for the inhibition of IL-6 bioactivity (50), BC response (51), and inflammatory cytokine production of human retinal endothelial cells (52). From the collective evidence, we conclude that the therapeutic effect in the TOFA group is very likely due to the immune-regulatory effect of TOFA rather than triamcinolone acetonide.

Currently, targeting JAK-associated pathways using JAK inhibitors such as TOFA has been increasingly applied clinically in various diseases (53). In animal models, TOFA increased Treg numbers and reduced Th17 cell counterparts (22, 54). Antiviral drugs combined with JAK inhibitors have yielded positive results for rapidly improving COVID-19 symptoms (55). Here, we first revealed the therapeutic effect of TOFA in VKH and comprehensively demonstrated its therapeutic mechanisms. The study demonstrated the heterogeneity of JAK-related genes, which were highly expressed in highly differentiated subsets. TOFA treatment inhibited the cytokine and JAK/STAT pathways in vitro and in vivo and rescued VKH-induced cell polarization and upregulated lymphocyte migration and activation. In addition, the inflammatory environment can result in Treg apoptosis and dampened antiinflammatory response (56). Notably, several apoptosis- and inflammation-related genes were inhibited by TOFA in CD4^+^ Tregs, indicating its therapeutic effect in reversing inflammatory damage caused by Tregs. TOFA also reduced IFN signaling and antigen presentation in BCs, which are important for BC and TC activation. In addition, PPI analysis of downregulated memory BC DEGs showed that IRF9 had the strongest correlation with other genes. Type I IFN-induced BC activation primarily induces IFN-stimulated genes via the JAK/STAT pathway involving complexes comprising IRF9 (27). Collectively, these results revealed cell-specific patterns in response to TOFA treatment and highlighted the therapeutic mechanisms of TOFA in TC and BC activation.

Human blood MYEs, including MCs and DCs, facilitate antigen presentation and inflammatory processes. The initiation and development of inflammatory responses, as in COVID-19 (57) and vascular inflammation (22), are closely linked to the key role of MCs. In autoimmune diseases, MCs have been regarded as the primary producers of inflammatory cytokines and mediate persistent inflammation, which involves the cytokine and JAK/STAT pathways (58–61). MCs from JAK2V617F-mutated myelofibrosis patients showed altered expression of chemokines and cytokine receptors (62). JAK inhibitors, including TOFA, had therapeutic effects in COVID-19 by suppressing cytokine storm progress related to MYEs (55, 57). However, the involvement of JAK/STAT pathway in MCs’ activation in VKH pathogenesis is unknown, and the impact of JAK targeting on a broader spectrum of MC states remains to be determined. We noticed that MCs, especially CMCs, which were most strongly affected by VKH and TOFA, exhibited an apparent inclination toward inflammation. In addition, VKH enhanced monocyte-lymphocyte interactions, characterized by increased cell activation and chemotaxis of MCs to other cells. This immune dysregulation was rescued by TOFA treatment. With the advantage of the single-cell method, we reclustered CMCs and identified 2 subgroups that were strongly associated with VKH disease and response to TOFA treatment. Of the 2 subclusters that were increased in patients with VKH and decreased after TOFA treatment, 1 had high inflammatory gene expression (*IL1B* and *IER2*), and the other had high JAK-related gene expression (*PIM1* and *STAT1*). The previous rationale for using TOFA to treat inflammatory diseases relied on the central role of TCs in disease pathogenesis and the effective inhibition of JAK inhibitors on TCs’ activation (21). Thus, we demonstrated the therapeutic effect of TOFA in patients with VKH and broadened our knowledge of the therapeutic mechanisms of TOFA treatment on circulating MCs.

Nevertheless, there were some limitations to our study. All patients included in this study have not received systemic glucocorticoid therapy before, while the patients in TOFA group were intolerant to systemic glucocorticoids. A small sample size was another one. Therefore, the findings might not be universally applicable to all types of patients with VKH.

In summary, we determined the therapeutic effect of TOFA on VKH disease and explored its action on the immune system using single-cell approaches. To delineate the pivotal cellular and molecular differences before and after TOFA treatment using CyTOF and scRNA-Seq, such as the JAK/STAT pathway in effector CD4^+^ TCs, BCs, and inflammatory MCs, we explored the potential contributions of TOFA in reducing inflammation, cellular polarization, and autoreactive signatures. The multimodal single-cell technique conducted in this study furnishes a comprehensive profile of the immune pathogenesis of VKH and insights into the feasibility and therapeutic mechanism of TOFA treatment for patients with VKH.

## Methods

### Human participants and ethics statement

To explore the therapeutic effect of TOFA as initial treatments in patients with VKH, we performed a retrospective study of 28 VKH patients (Supplemental Table 1). All patients were diagnosed based on the disease manifestations, and the results of standard OCT and fluorescein angiography, according to the Revised Diagnostic Criteria of VKH (63). Only patients first diagnosed with VKH who had not received systematic treatment were reviewed. Medical records of patients with VKH treated with conventional therapy (refers to systemic glucocorticoids plus peribulbar injection of triamcinolone acetonide) or systemic tofacitinib therapy plus peribulbar injection of triamcinolone acetonide at 3 months of follow-up were analyzed. In the conventional therapy group, 18 patients with VKH were enrolled, and glucocorticoids were administrated from an initial oral 1.2 mg/kg and a gradually decreased dose combined with a peribulbar injection of triamcinolone acetonide (20 mg) monthly. In the TOFA group, 10 individuals diagnosed with VKH had not previously received systemic conventional therapy because they could not tolerate glucocorticoids. Tofacitinib was orally administered at 5 mg, twice a day, combined with a peribulbar injection of triamcinolone acetonide (20 mg) monthly. No remarkable complications were observed in either group. Eyesight was tested, and an OCT examination was performed every month to assess disease severity. In addition, 5 healthy participants who met the following criteria were included for the study: physical and psychological health; no clinically significant abnormalities in blood chemistry; and no medication, smoking, or obesity.

### Study protocol of single-cell analysis

Blood samples of patients with VKH were obtained at the day before and 1 month after starting tofacitinib treatment. Five samples each from the HC group, VKH group (before TOFA treatment), and TOFA group (after TOFA treatment) were included for single-cell analysis. PBMCs were isolated by standard density gradient centrifugation. Trypan blue (Solarbio) was used to identify the viability and quantity of PBMCs; cell viability of all samples exceeded 90% with more than 1 × 10^7^ viable cells. A proportion of PBMCs was allocated for scRNA-Seq analysis, and another was used for mass cytometry. To elucidate the impacts of VKH and TOFA on blood immune cells, we measured single-cell protein expression using a 39-marker CyTOF panel and RNA level using scRNA-Seq (*n* = 15; Supplemental Tables 2–4). By combining CyTOF and scRNA-Seq, we created a comparative atlas detailing the impact of VKH and TOFA on cell type distribution, gene expression changes, and cell-cell interactions.

### scRNA-Seq

#### scRNA-Seq data alignment, processing, and sample aggregation.

Single-cell suspensions were transformed into barcoded scRNA-Seq libraries using the Chromium Single Cell 5′ Library (10x Genomics, Genomics Chromium platform; Illumina NovaSeq 6000), Gel Bead and Multiplex Kit, and Chip Kit (10x Genomics). According to the manufacturer’s instructions, single-cell RNA libraries were prepared using the Chromium Single Cell 5′ v2 Reagent Kit (120237; 10x Genomics). FastQC software was used to check the quality of the library. Sequencing data were initially processed using CellRanger software (version 4.0; 10x Genomics). The count pipeline in the CellRanger Software Suite was used to demultiplex and barcode the sequences. Based on the calculation of the single-cell expression matrix by CellRanger, filtration, normalization, linear dimensionality reduction, selecting the dimension of dimensionality reduction for subsequent analysis, clustering, nonlinear dimensionality reduction visualization, and differential gene expression analysis were performed using the Seurat package (version 3.0). We removed the cell population that expressed HBB, HBA1, and several light and heavy chain transcripts, which are considered erythrocyte contamination, before filtration using the Seurat package. Next, cells with fewer than 200 detected genes and a mitochondrial gene ratio greater than 15% were excluded. After quality control, a total of 15 libraries were sequenced, and 160,176 cells (HC: 59,350 cells; VKH: 54,720 cells; TOFA: 46,106 cells) were analyzed in subsequent studies.

#### Dimensionality reduction and clustering analysis.

The “NormalizeData” function was used to logarithmically normalize the counts per cell (1 + counts per 10,000). The “FindVariableGenes” function in Seurat extracted the top 10 most variable genes with the default parameters. Dimensionality was implemented with the “RunPCA” function. Significant clusters were identified by the “FindNeighbors” and “FindClusters” functions at an appropriate resolution. The 2-dimensional t-SNE algorithm based on “RuntSNE” function was used to visualize cells. Marker genes of each significant cluster were identified by “FindAllMarkers” function.

#### Differential expression analysis.

For each cell type between different groups, the Wilcoxon rank-sum test implemented in the “FindMarkers” function of the Seurat package (version 3.0) was used for differential expression analysis. After identifying DEGs after pairwise comparison, VKH-related DEG data set and VKH-related DEGs were filtered and established (adjusted *P* < 0.05, |log_2_ fold change| > 0.25).

#### Analysis of gene functional enrichment.

The Metascape web tool (64) was used to perform GO biological process and pathway analysis, and PPIs, which allowed us to visualize the functional patterns of DEGs and conduct statistical analysis. We visualized the top 10 of 30 VKH- or TOFA-related terms using the ggplot2 R package. In addition, we performed the drug discovery bioinformatics analysis based on the Metascape web tool. After gene annotation of DisGeNET and DrugBank databases, 16 uveitis-associated genes and their targeted drugs were screened to predict other potential therapeutics for VKH treatment (Supplemental Table 6).

#### Scoring of biological processes.

By calculating the mean normalized expression of corresponding genes, individual cells were scored for their expression of gene signatures that represented certain biological functions. Functional signature with the full gene list is provided in Supplemental Table 7. For instance, the inflammatory response score was measured by calculating the mean expression of genes in the GO term inflammatory response (GO: 0006954). Genes related to JAK/STAT pathway were obtained from the Kyoto Encyclopedia of Genes and Genomes pathway data set Jak Stat Signaling Pathway (PW_0000209).

#### Determination of cell-cell interactions.

With the help of CellChat (https://github.com/sqjin/CellChat; commit ID 9e1e605) R package and iTALK (https://github.com/Coolgenome/iTALK; commit ID 6d9b390), cell-cell communication between different cells was predicted based on the scRNA-Seq data. iTALK tool was also applied to analyze and visualize the differences in cellular communication between different groups. If the ligands and receptors were not detected, communication was considered absent. Thus, VKH-specific cell-cell L-R pairs, which were only detected in VKH group, were selected. TBTools (https://www.tbtools.com) was used to normalize the data and draw a heatmap. In addition, we used CellChat R package to analyze and visualize signaling pathway networks among groups with default parameters.

### Mass cytometry

#### Antibodies and reagents.

Monoclonal anti-human antibodies for mass cytometry were either acquired preconjugated with heavy metal isotopes (Fluidigm) or conjugated via the Maxpar X8 Chelating Polymer Kit (Fluidigm). The 39 antibodies were used to recognize immune cells and detect the markers’ expression (Supplemental Table 4). The following steps were adapted from the published article (65).

#### Live-cell barcoding and surface staining.

To decrease intersample staining variability, sample processing time, and antibody consumption, a live-cell barcoding methodology was applied. A total of 0.5 μmol/L viability dye (cisplatin-195pt; 201064; Fluidigm) was applied to stain the barcoded and combined samples, which were vortexed for 2 minutes at room temperature (RT). Then we terminated the reaction with Maxpar Cell Staining Buffer (Fluidigm) on a rotating shaker (400 rcf) at RT. Then, on a rotary shaker (500 rpm), 1.6% paraformaldehyde in PBS was used to wash and fix the cells for 10 minutes at RT. To slow the fixation reaction, precooled Maxpar Cell Staining Buffer was applied to resuspend the cells, which were then washed twice with PBS/bovine serum albumin and once with double-distilled water. Finally, we resuspended the cells in 400 μL surface antibody mixture and incubated at 37°C for 30 minutes on a rotating shaker (500 rpm) for surface staining. Then the samples were stored overnight at 4°C in freshly diluted 2% formaldehyde in PBS that contained 0.125 nmol/L iridium 191/193 intercalator (Fluidigm, 201192).

#### Intracellular factor staining.

The cells were washed twice with permeabilization buffer (0.5% saponin, 2% bovine serum albumin, and 0.01% sodium azide [all MilliporeSigma] in PBS). Then we resuspended the cells in 50 μL of intracellular antibody mixture in permeabilization buffer for 1 hour on a rotary shaker at 4°C. Then we washed the samples, removed the supernatant, and resuspended the cells in 1× iridium intercalator solution (Fluidigm) overnight. At last, the sample was washed twice with PBS/bovine serum albumin and once with double-distilled water before acquisition.

#### Mass cytometry acquiring, processing, and quality control.

At an event rate of less than 400/s, CyTOF data were acquired from a SuperSampler fluidics CyTOF2 system (Victorian Airships), then normalized with the help of Helios normalizer software (version 6.7.1016; Fluidigm). The CyTOF2 mass cytometer (Fluidigm) was quality controlled and tuned daily. Cytobank software (version 7.0; https://mtsinai.cytobank.org) was applied to deconvolute barcoded samples and filter cross-sample doublets. Cytobank was applied to sequentially remove calibration beads, dead cells, debris, and barcodes of CD45^+^ PBMCs based on event length and live cell (195Pt) and DNA (191Ir and 193Ir) channels. Then, the FCS files were exported for downstream analysis. All cytometry data were converted to an inverse hyperbolic sine (arcsinh) function (mass cytometry: cofactor of 5) using R.

#### Mass cytometry visualization and clustering.

FlowCore R package was used to read and process the FCS files for further analysis. For samples with over 12,000 cells, we selected 12,000 cells randomly to ensure that samples were equally representative. The CATALYST R package was applied to integrate data for analysis. To identify specific populations, all FlowSOM R package–based clustering and subclustering were performed on the data set. Mass cytometry data sets from all individuals of each cell type were created for analysis. The detailed cell counts are provided in Supplemental Table 8.

### Flow cytometry of PBMCs in vitro

After isolation, PBMCs (3 × 10^5^) were cultured in the presence of anti-CD3/CD28 in a 1:1 ratio and treated with TOFA (0.08–2 μM) for 2 days. To investigate the intercellular cytokines in TCs, hemocytes were stimulated by a leukocyte activator, incorporating ionomycin (500 ng/mL; MilliporeSigma) and PMA (50 ng/mL; MilliporeSigma), then inhibited by brefeldin A (1 μg/mL; MilliporeSigma) in a 5% CO_2_ environment at 37°C for 4 hours. Thereafter, processed cells were stained with anti-human CD3 Brilliant Violet 421 (BioLegend), anti-human CD8 PE-Cy7 (BioLegend), and anti-human IFN-γ APC (BD Biosciences) for Th1 cell analysis; anti-human IL-17A PE (BioLegend) for Th17 cell analysis; and anti-human FOXP3 PE (BioLegend) for Treg analysis. The labeled cells were measured using a BD Biosciences LSR Fortessa flow cytometer. Ultimately, the harvested data were analyzed with FlowJo software (version 10.0; TreeStar).

### Phosphospecific flow cytometry (phospho-flow analysis)

PBMCs (3 × 10^5^) were pretreated with TOFA (0.08–2 μM) and cultured in the presence of anti-CD3/CD28 in a 1:1 ratio for 2 days. Then the cells were stimulated using IL-2 (200 U/mL) for 30 minutes. The cells were fixed for 20 minutes at RT with 4% paraformaldehyde. Washed cells were permeabilized in ice-cold 75% methanol for 30 minutes and simultaneously incubated with the following antibodies: anti-human CD3 Brilliant Violet 421, CD8 PE-Cy7, p-STAT1 (Tyr701) PE, and p-STAT3 (Tyr705) Alexa Fluor 488 (BioLegend). Flow cytometry analysis was performed using a BD Biosciences LSR Fortessa flow cytometer. Ultimately, the harvested data were analyzed with FlowJo software (version 10.0 TreeStar). STAT phosphorylation was appraised in CD3^+^ and CD8^+^ TCs.

### Cell viability assay

PBMCs (5 × 10^3^/well) were seeded into a 96-well plate, treated with various concentrations of TOFA (0.08–10 μM), and incubated at 37°C in a humidified incubator under 5% CO_2_ for 24 hours. Cell viability was determined using a Cell Counting Kit-8 (CCK8), according to the manufacturer’s protocol.

### RNA isolation and real-time quantitative PCR

Total RNA from PBMCs was extracted by TRIzol reagent (Invitrogen), then quantified with a NanoDrop spectrophotometer (NanoDrop Technologies). Next, cDNAs were synthesized using the PrimeScript RT Master Mix (Perfect Real Time, TaKaRa Bio Inc.). Real-time quantitative PCR was performed using SYBR Premix Ex Taq II (TaKaRa Bio Inc.) in accordance with the manufacturer’s instructions. Primer sequences were as follows: human *GAPDH* (Forward: 5′-TCGGAGTCAACGGATTTGGT-3′, Reverse: 5′-TTCCCGTTCTCAGCCTTGAC-3′); human *TNFAIP3* (Forward: 5′-CACGCTCAAGGAAACAGACA-3′, Reverse: 5′-CATGGGTGTGTCTGTGGAAG-3′); human *NOD1* (Forward: 5′-CAGAGTCTCACCCCCACATT-3′, Reverse: 5′-CGGCCGAGAAGTAGTCATTC-3′); human *NOD2* (Forward: 5′-GCGCGATAACAATATCTCAGA-3′, Reverse: 5′-CAGAGTTCTTCTAGCATGACG-3′). The relative mRNA levels of *TNFAIP3*, *NOD1*, and *NOD2* were determined using the 2–ΔΔCt method, and the level of *GAPDH* mRNA was used as a loading control.

### ELISA analysis

After PBMC isolation, sera were collected from the samples and stored at –80°C for further analysis. Serum TNF-α levels were determined using an ELISA kit (88-7346-88; Invitrogen).

### Data availability

The scRNA-Seq data are deposited in the Genome Sequence Archive in BIG Data Center, Beijing Institute of Genomics (https://bigd.big.ac.cn/gsa-human/), Chinese Academy of Sciences, under the Project Accession No. PRJCA009601 and GSA Accession No. HRA002400. Experimental protocols and the data analysis pipeline used in scRNA-Seq follow those described on the 10x Genomics and Seurat official websites.

### Statistics

The cell percentages between the VKH and HC groups and TOFA and VKH groups were compared using 2-tailed unpaired Student’s *t* test and 2-tailed paired *t* tests, respectively, and data analysis and presentation were performed using GraphPad Prism (version 8.0.2; GraphPad Software Inc.). To compare the markers or genes at protein levels among groups, a 2-sided Wilcoxon test, which was accomplished in the function “compare_means” of the ggpubr R package using default parameters, was used to calculate the *P* value. When computing the GO biological process and pathway, *P* values were obtained through a hypergeometric test with default parameters in the Metascape web tool. Details on the size of biological replicates and assays are provided in each figure legend. *P* < 0.05 was considered statistically significant.

### Study approval

The study was registered in the clinical trial (ID: ChiCTR2000030237) and approved by the Ethics Committee of Zhongshan Ophthalmic Center, Guangzhou, China (ID: 2020KYPJ124). Written informed consent was obtained from all participants, and all procedures were performed according to the International Ethical Guidelines for Research Involving Human Subjects, as stated in the Declaration of Helsinki.

## Author contributions

WS and YZ designed the study; XL, QJ, and JL led the bioinformatic analyses; XL and SY performed the experiments; XL, QJ, JL, and SY took care of participants and provided the clinical information; ZH, RD, TT, ZL, and RJ provided intellectual input and comments into this study; and XL, QJ, and JL wrote the paper. All authors have read and approved the final manuscript.

## Supplementary Material

Supplemental data

Supplemental tables 1-8

## Version 1 10/27/2022

In-Press Preview

## Version 2 12/08/2022

Electronic publication

## Version 3 01/07/2025

The Acknowledgments section was updated
