# Supplementary material for: Insights gained from single-cell analysis of immune cells in tofacitinib treatment of Vogt-Koyanagi-Harada disease
Source: JCI Insight. 2022 Dec 8;7(23):e162335. doi: 10.1172/jci.insight.162335 (PMC9746911; doi:10.1172/jci.insight.162335)
Supplement: Supplemental data [file jciinsight-7-162335-s010.pdf]

Supplemental Material for

Insights gained from single-cell analysis of immune cells in Tofacitinib treatment of Vogt-Koyanagi-Harada disease

Xiuxing Liu[1, 2], Qi Jiang[1, 2], Jianjie Lv[1, 2], Shizhao Yang[1, 2], Zhaohao Huang[1], Runping Duan[1], Tianyu Tao[1], Zhaohuai Li[1], Rong Ju[1], Yingfeng Zheng[1,*], Wenru Su[1,*]

[1] State Key Laboratory of Ophthalmology, Zhongshan Ophthalmic Center, Sun Yat-sen University, Guangdong Provincial Key Laboratory of Ophthalmology and Visual Science, Guangzhou 510060, China

[2] These authors contributed equally

*Corresponding authors: Wenru Su and Yingfeng Zheng

Email: suwr3@mail.sysu.edu.cn (W.S.); zhyfeng@mail.sysu.edu.cn (Y.Z.)

Figures S1 to S7

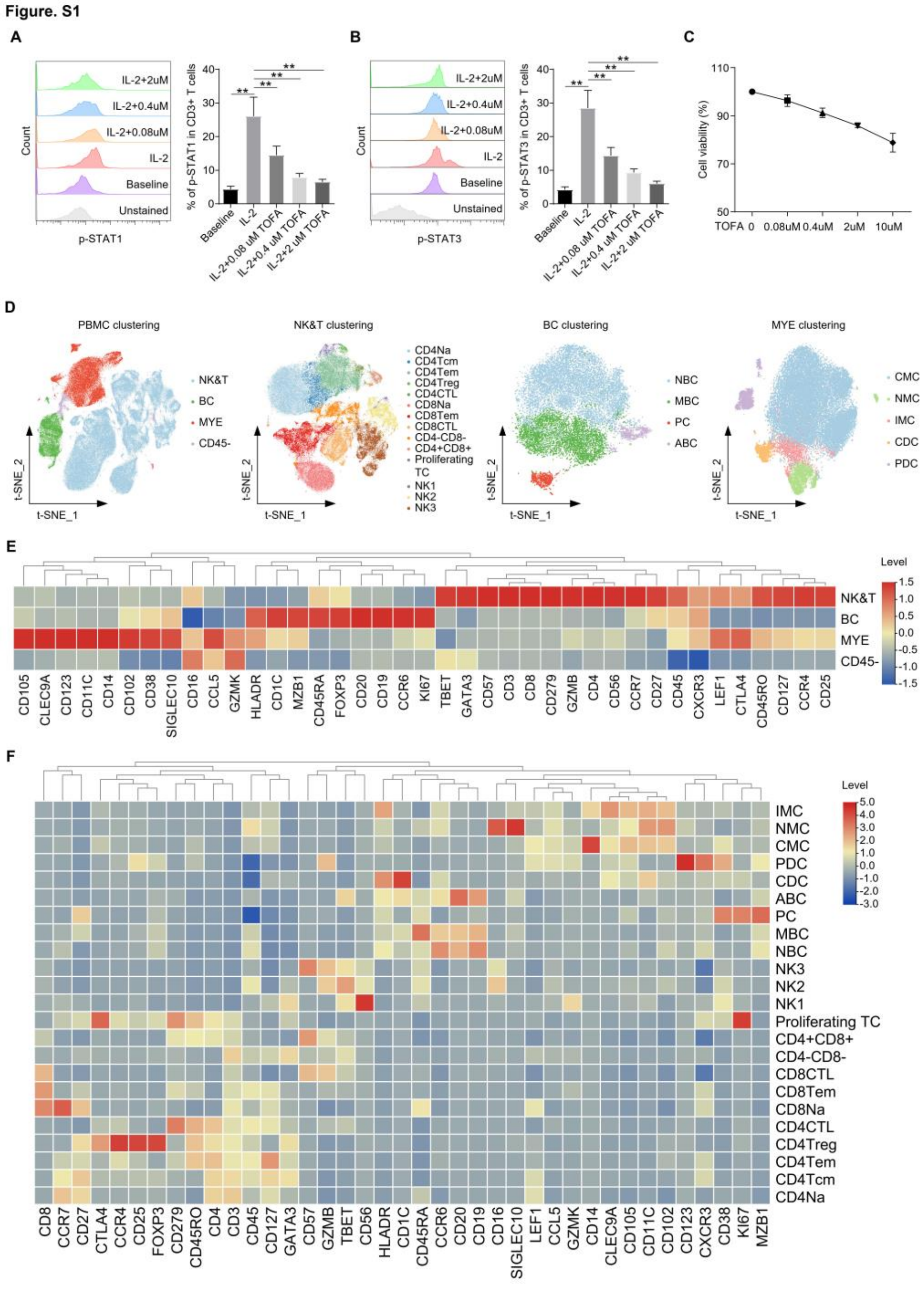

Figure S1. The inhibitory effects of TOFA on PBMC in vitro and clustering strategy in CyTOF.

The flow cytometry histograms (left) and box plots (right) showing the phosphorylation of STAT1 (**A**) and STAT3 (**B**) in CD3+ T cells (n = 6/group).

C. The cytotoxicity of tofacitinib on PBMCs (n = 4/group).

The clustering strategy of major immune cell populations (**D**) identifying 4 PBMC types based on the scaled expression heatmap of discriminative gene for each cluster (**E**). Then NK&T, BC and MYE (**D**) were re-clustered and identified classical subsets based on the scaled expression heatmap of discriminative gene for each cluster (**F**).

Significance in **A-B** was calculated using two-tailed unpaired Student's t-test; **P < 0.01.

Figure. S2

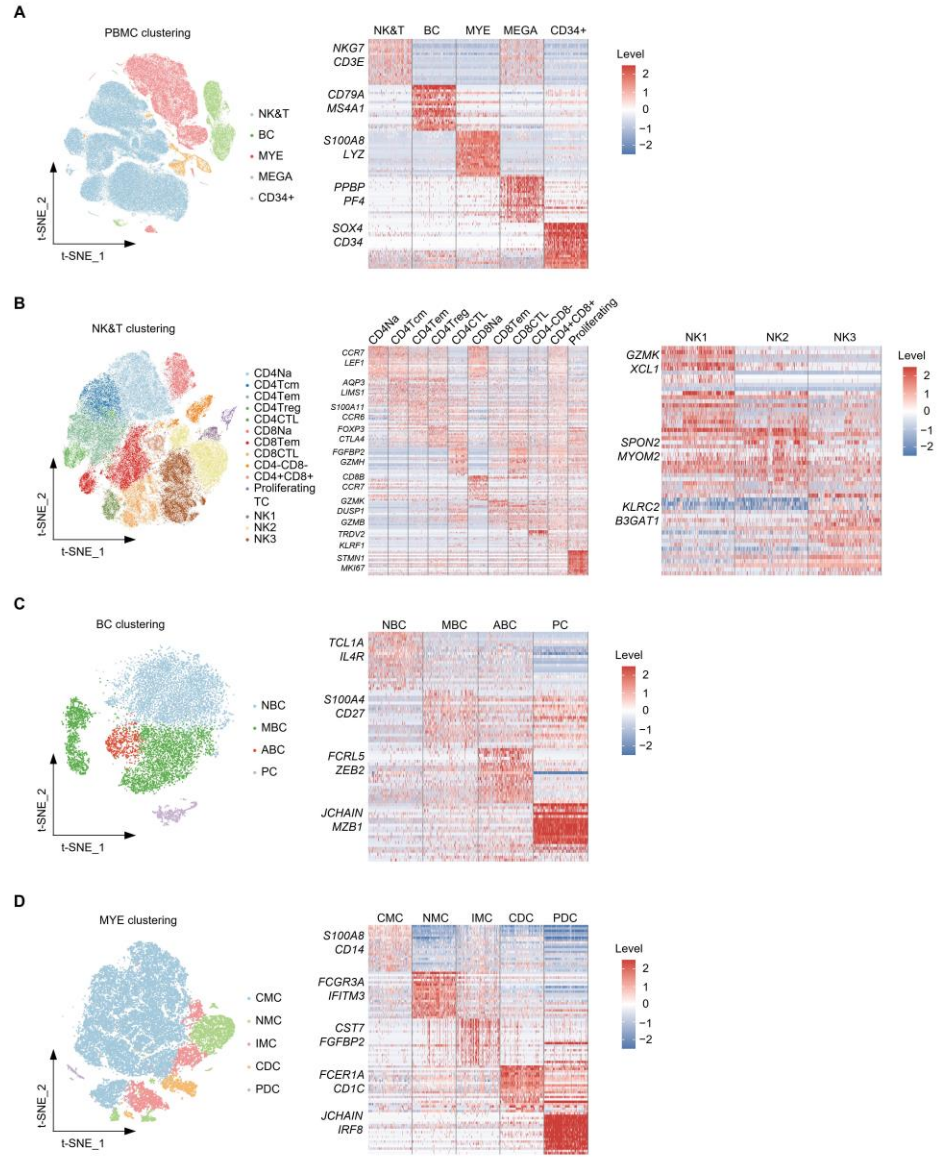

Figure S2. Clustering strategy in scRNA-seq.

The clustering strategy of major immune cell populations (**A**) identifying 5 PBMC types (left) based on the scaled expression heatmap of discriminative gene for each cluster (right). Then NK&T (**B**), BC (**C**) and MYE (**D**) were re-clustered and identified classical subsets (left) based on the scaled expression heatmap of discriminative gene for each cluster (right). Color scheme is based on z-score distribution from −2 (blue) to 2 (red).

The full names of the cell types in CyTOF and scRNA-seq are as follows: NK&T, NK and T cells; BC, B cells; MYE, myeloid cells; CD4Na, CD4+ naive T cell; CD4Tcm, central memory CD4+ T cell; CD4Tem, effector memory CD4+ T cell; CD4Treg, regulatory CD4+ T cell; CD4CTL, cytotoxic CD4+ T cell; CD8Na, CD8+ naive T cell; CD8Tem, effector memory CD8+ T cell; CD8CTL, cytotoxic CD8+ T cell; CD4-CD8-, CD4-CD8- double-negative T cell; CD4+CD8+, CD4+CD8+ double-positive T cell; Proliferating TC, proliferating T cell; NK1, CD16 (*FCGR3A)*- CD56(*NCAM1)*^{bright} NK; NK2, CD16(*FCGR3A)*+ CD56*(NCAM1)*^{dim} CD57(*B3GAT1)*- NK; NK3, CD16(*FCGR3A)*+ CD56(*NCAM1)*^{dim} CD57(*B3GAT1)*+ late NK; NBC, naive B cell; MBC, memory B cell; ABC, autoimmune-associated B cell; PC, plasma cell; CMC, classical monocyte; NMC, nonclassical monocyte; IMC, intermediate monocyte; CDC, conventional DC; PDC, plasmacytoid DC; CD45-, CD45- cells; MEGA, megakaryocyte; CD34+, CD34+ cells.

Figure. S3

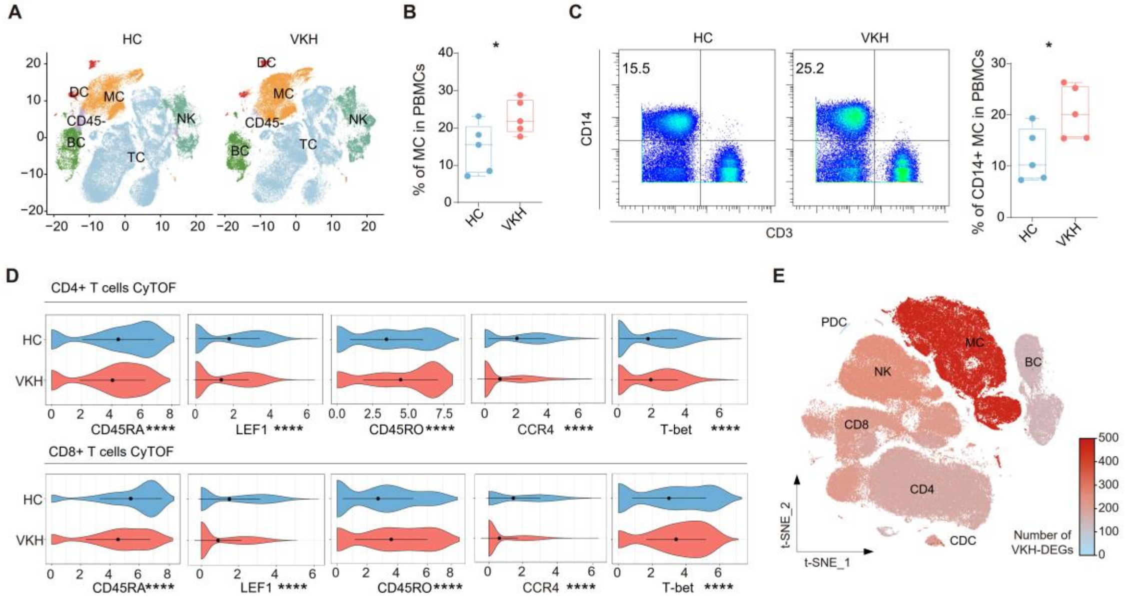

Figure S3. Reconstitution of the circulating cellular ecosystem by VKH.

A. t-SNE plot of immune cells in HC and VKH groups.

B. The percentage of MC in PBMCs between HC and VKH groups (n = 5/group).

C. The flow cytometry histogram showing the expression of CD3 and CD14, and the percentage of CD14+ MC in PBMCs between HC and VKH groups (n = 5/group).

D. Violin plot showing the expression of CD45RA, LEF1, CD45RO, CCR4 and T-bet in CD4+ or CD8+ T cells between HC and VKH groups in CyTOF.

E. t-SNE plot of immune cells, and the color indicating the number of VKH-DEGs.

Significance in **B**, **C** was calculated using two-tailed unpaired Student's t-test; *P < 0.05, ****P < 0.0001.

Figure. S4

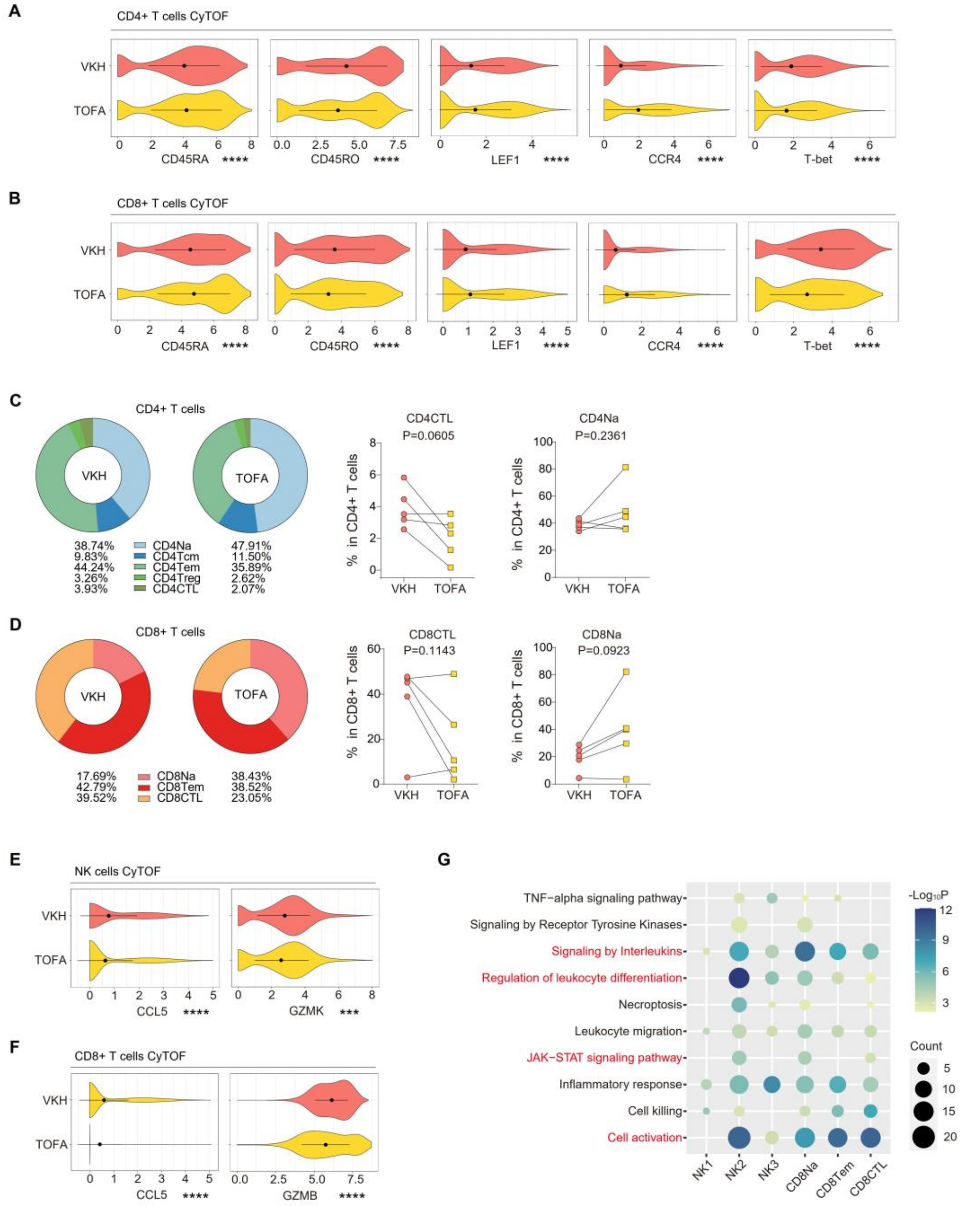

Figure S4. TOFA treatment downregulated cell activation of NKs and TCs.
Violin plot showing the expression of CD45RA, CD45RO, LEF1, CCR4 and T-bet in CD4+(**A**) or CD8+(**B**) T cells between VKH and TOFA groups in CyTOF.
C. The percentage of CD4+ TCs subsets in CD4+ T cells in VKH and TOFA groups (n = 5/group).
D. The percentage of CD8+ subsets in CD8+ TCs in VKH and TOFA groups (n = 5/group).
E. Violin plot showing the expression of CCL5 and GZMK in NK cells between VKH and TOFA groups in CyTOF.
F. Violin plot showing the expression of CCL5 and GZMB in CD8+ T cells between VKH and TOFA groups in CyTOF.
G. Representative GO biological process and pathways enriched in downregulated TOFA-DEGs based on functional enrichment analysis in NK and CD8+ TCs subsets.
Significance in **C-D** was calculated using two-tailed paired t-test; ****P < 0.0001.

Figure. S5

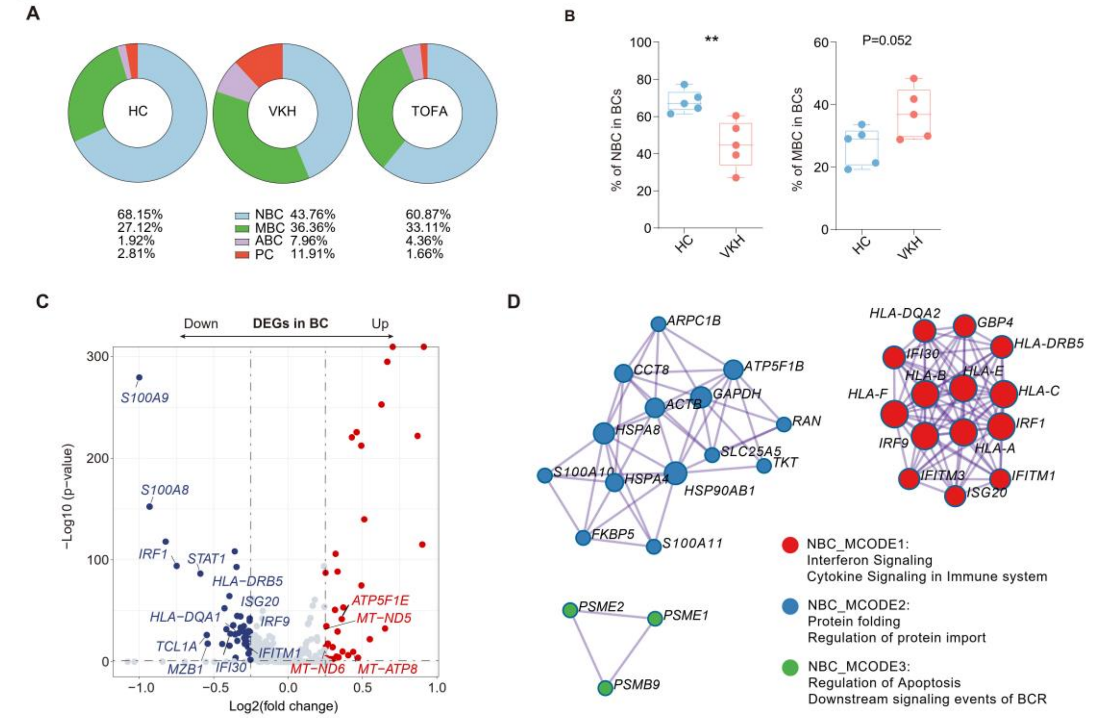

Figure S5. TOFA treatment downregulated cell activation of BCs.
A. The pie plots showing the percentage of BCs subsets in HC, VKH and TOFA groups.
B. The percentage of NBC and MBC in BCs between HC and VKH groups (n = 5/group).
C. Volcano plot showing TOFA-DEGs in BC.
D. The gene network showing PPI analysis of downregulated TOFA-DEGs in NBC subset.
Significance in **B** was calculated using two-tailed unpaired Student's t-test; **P < 0.01.

Figure. S6

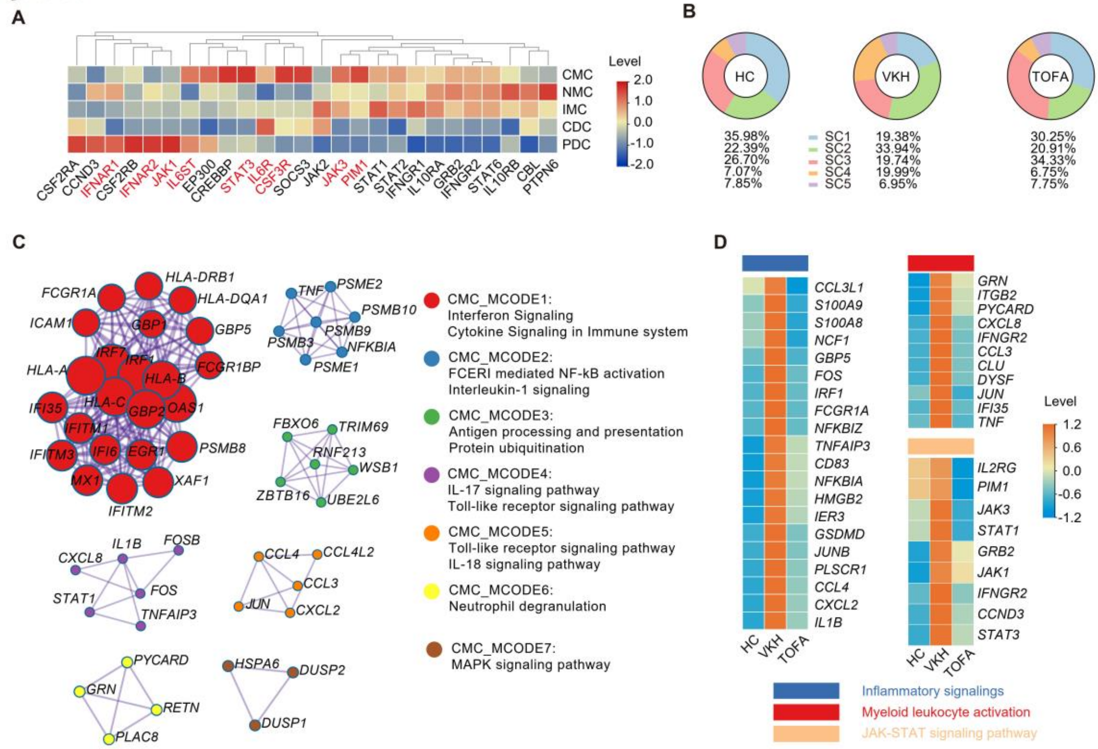

Figure S6. TOFA treatment downregulated cell activation of myeloid cells.
A. The expression levels of genes related to JAK-STAT signaling in myeloid cells subsets.
B. The pie plots showing the percentage of CMCs-SCs in HC, VKH and TOFA groups.
C. The gene network showing PPI analysis of downregulated TOFA-DEGs in CMC subset.
D. The heatmap showing the levels of genes associated with specific pathways in CMC among HC, VKH and TOFA groups.

Figure. S7

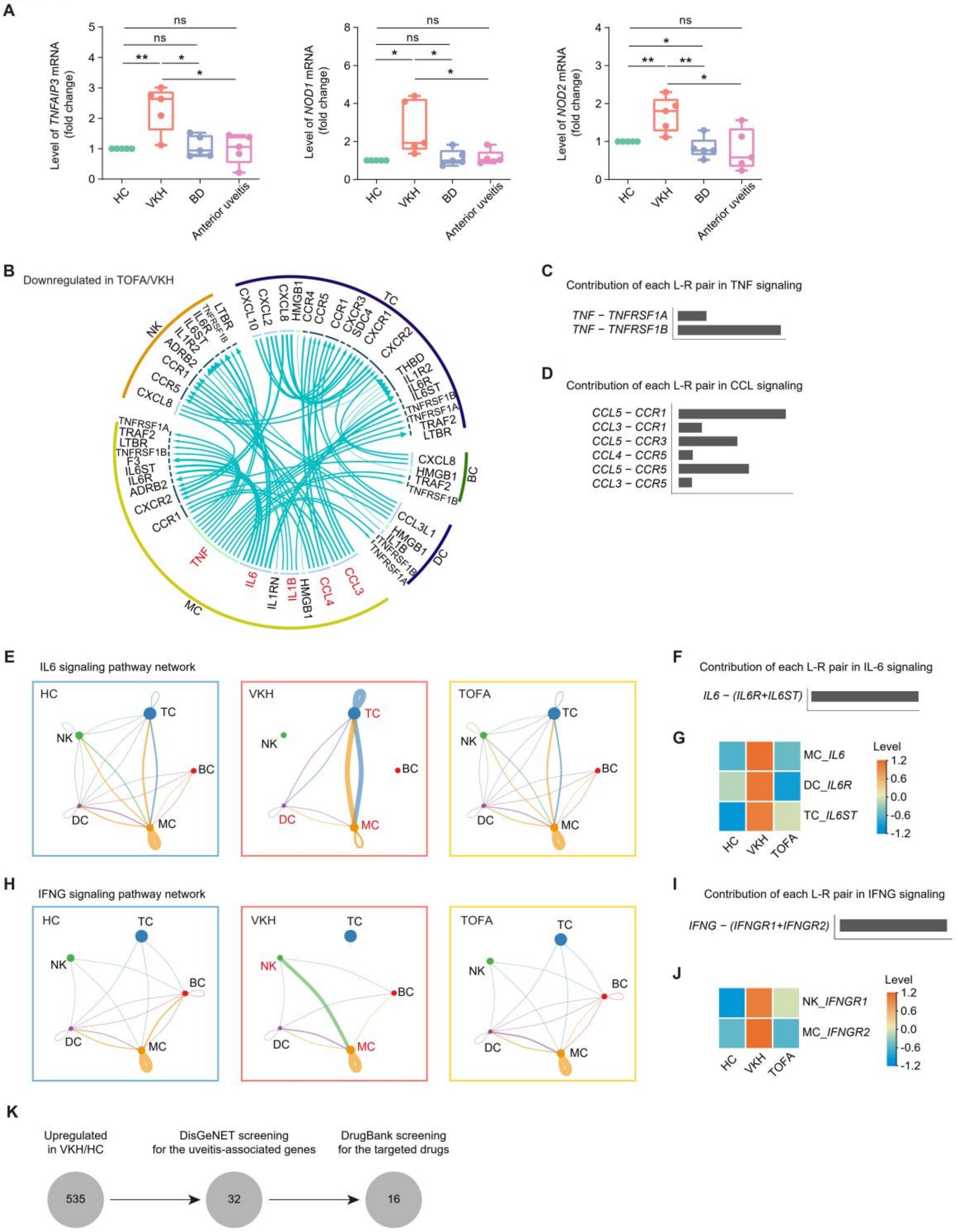
A
Level of TNFAIP3 mRNA (fold change)
Level of NOD1 mRNA (fold change)
Level of NOD2 mRNA (fold change)
HC
VKH
BD
Anterior uveitis
ns
**
*
B
Downregulated in TOFA/VKH
NK
TC
BC
DC
MC
C
Contribution of each L-R pair in TNF signaling
TNF − TNFRSF1A
TNF − TNFRSF1B
D
Contribution of each L-R pair in CCL signaling
CCL5 − CCR1
CCL3 − CCR1
CCL5 − CCR3
CCL4 − CCR5
CCL5 − CCR5
CCL3 − CCR5
E
IL6 signaling pathway network
HC
VKH
TOFA
F
Contribution of each L-R pair in IL-6 signaling
IL6 − (IL6R+IL6ST)
G
MC_IL6
DC_IL6R
TC_IL6ST
Level
H
IFNG signaling pathway network
I
Contribution of each L-R pair in IFNG signaling
IFNG − (IFNGR1+IFNGR2)
J
NK_IFNGR1
MC_IFNGR2
Level
K
Upregulated in VKH/HC
DisGeNET screening for the uveitis-associated genes
DrugBank screening for the targeted drugs
535
32
16

Figure S7. TOFA treatment reduced the cell-cell interaction influenced by VKH.

A. The mRNA expression levels of *TNFAIP3*, *NOD1*, and *NOD2* were measured with real-time quantitative PCR (n = 5/group).

B. Circle plot showing the downregulated L-R pairs in in TOFA/VKH comparison.

C. Relative contribution of each L-R pair in TNF signaling.

D. Relative contribution of each L-R pair in CCL signaling.

E. Circle plot showing IL6 signaling pathway network in HC, VKH and TOFA groups.

F. Relative contribution of each L-R pair in IL-6 signaling.

G. The heatmap showing the levels of MC-related *IL6*, DC-related *IL6R* and TC-related *IL6ST* among HC, VKH and TOFA groups.

H. Circle plot showing IFNG signaling pathway network in HC, VKH and TOFA groups.

I. Relative contribution of each L-R pair in IFNG signaling.

J. The heatmap showing the levels of NK-related *IFNGR1* and MC-related *IFNGR2* among HC, VKH and TOFA groups.

K. Schematic of the drug discovery bioinformatics analysis.

Significance in **A** was calculated using two-tailed unpaired Student's t-test; *P < 0.05, **P < 0.01.
